# Spin-Symmetry Breaking and Hyperfine Couplings in Transition-Metal Complexes Revisited Using Density Functionals Based on the Exact-Exchange Energy Density

**DOI:** 10.1021/acs.jctc.3c01422

**Published:** 2024-02-27

**Authors:** Artur Wodyński, Bryan Lauw, Marc Reimann, Martin Kaupp

**Affiliations:** Technische Universität Berlin, Institut für Chemie, Theoretische Chemie/Quantenchemie, Sekr. C7, Straße des 17. Juni 135, Berlin, D-10623, Germany

## Abstract

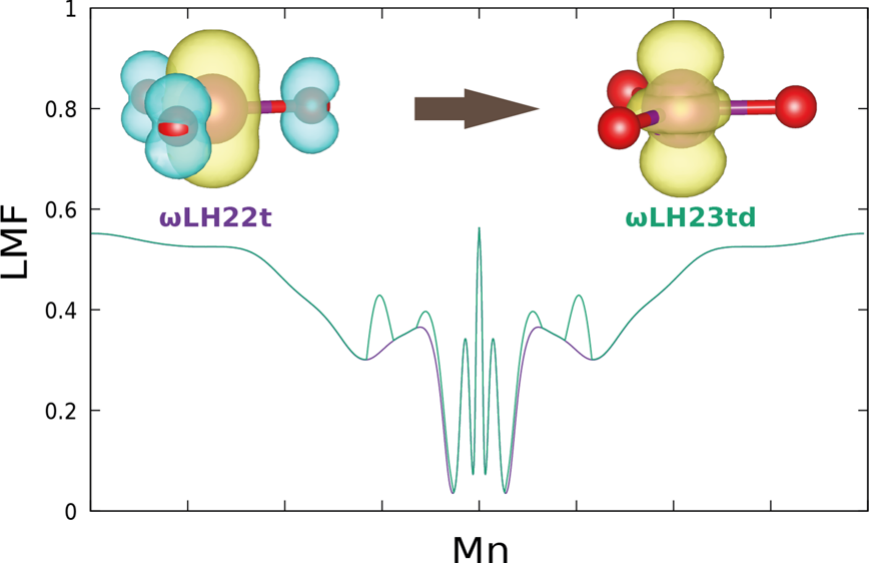

A small set of mononuclear manganese complexes evaluated previously for their Mn hyperfine couplings (HFCs) has been analyzed using density functionals based on the exact-exchange energy density—in particular, the spin symmetry breaking (SSB) found previously when using hybrid functionals. Employing various strong-correlation corrected local hybrids (scLHs) and strong-correlation corrected range-separated local hybrids (scRSLHs) with or without additional corrections to their local mixing functions (LMFs) to mitigate delocalization errors (DE), the SSB and the associated dipolar HFCs of [Mn(CN)_4_]^2–^, MnO_3_, [Mn(CN)_4_N]^−^, and [Mn(CN)_5_NO]^2–^ (the latter with cluster embedding) have been examined. Both strong-correlation (sc)-correction and DE-correction terms help to diminish SSB and correct the dipolar HFCs. The DE corrections are more effective, and the effects of the sc corrections depend on their damping factors. Interestingly, the DE-corrections reduce valence-shell spin polarization (VSSP) and thus SSB by locally enhancing exact-exchange (EXX) admixture near the metal center and thereby diminishing spin-density delocalization onto the ligand atoms. In contrast, sc corrections diminish EXX admixture locally, mostly on specific ligand atoms. This then reduces VSSP and SSB as well. The performance of scLHs and scRSLHs for the isotropic Mn HFCs has also been analyzed, with particular attention to core–shell spin-polarization contributions. Further sc-corrected functionals, such as the KP16/B13 construction and the DM21 deep-neural-network functional, have been examined.

## 1 Introduction

Open-shell transition-metal complexes are important in many chemical and biochemical processes, and their quantum-chemical description is therefore a central research area. Yet, such compounds are often difficult to treat quantum-chemically.^1−3^ In particular, complexes of 3d metal centers introduce substantial challenges for describing electron correlation. This is in part due to the lack of a radial node of the 3d-shell, which leads to a small radial extent. Substantial Pauli repulsion of metal–ligand or metal–metal bonding orbitals with the 3s/3p semicore–shells at the metal center results, as these have a similar radial extent.^4^ This leads essentially to stretched bonds in many cases, thereby introducing substantial static correlation. Indeed, due to this stretched-bond situation, 3d–3d metal–metal bonds often exhibit charge-shift bonding.^5^

As the extent of static correlation is highly variable, and an adequate inclusion of dynamical correlation is important as well, either single-reference or multireference post-Hartree–Fock wave function approaches are considered as the appropriate tools for open-shell transition-metal complexes, depending on the system of interest. However, as such methods tend to exhibit very steep scaling with system size, the vast majority of quantum-chemical calculations, even in this challenging subfield of transition-metal chemistry, is done using Kohn–Sham density-functional theory (KS DFT). DFT methods can typically be applied routinely to much larger systems, but the accuracy of DFT calculations depends crucially on the choice of approximate exchange-correlation (XC) functional, i.e., on the specific density-functional approximation (DFA). While theoretically the exact XC functional should also be able to address systems exhibiting multireference situations, the suitability of different existing DFAs is not a priori clear. Since the noninteracting reference system of KS DFT is typically described by a single Slater determinant, the validity of the so-called adiabatic connection (AC) linking the reference system with the fully interacting system while keeping the electron density constant can be called into question.^6^ This applies in particular when the presence of strong correlations would suggest the fully interacting system to have clear multideterminantal character. At the same time, most DFAs suffer from self-interaction errors (SIE), which typically render metal–ligand bonds too covalent and thereby delocalize too much spin density between the metal and the ligand (“delocalization errors” (DEs)).^7,8^ To reduce simultaneously static correlation (sc) errors (often expressed in terms of fractional spin errors (FSEs)^9,10^) and DEs (often expressed in terms of fractional charge errors (FCEs)^11,12^) is arguably the outstanding challenge^13,14^ in the contemporary development of DFAs. The fact that a larger admixture of exact exchange (EXX) in hybrid functionals tends to improve on FCEs but is detrimental for FSEs, and vice versa, has been termed the “zero-sum game” of DFAs.^15,16^

We have recently introduced density functionals depending on the EXX energy density, which include corrections for sc.^17−20^ More specifically, we have applied sc factors in the spirit of the B13^21^ and KP16/B13^22^ coordinate-space models for nondynamic and strong correlations to local hybrid functionals (scLHs)^17−19^ and to range-separated local hybrids (RSLHs),^20^ generating many scLHs and strong-correlation range-separated local hybrids (scRSLHs). These show substantial improvements over the underlying LHs and RSLHs regarding spin-restricted bond-disssociation curves and the closely related FSEs of open-shell atoms. While we have also already found appreciable improvements for more “real-world” systems, including the magnetizabillities of molecules like ozone,^23^ further evaluations of these new functionals are mandatory to judge their overall usability in challenging chemical systems. Naturally, this should also be done for open-shell transition-metal complexes.

In this work, we will use such advanced functionals to re-examine the so-called “spin-polarization/spin-contamination” dilemma of transition-metal hyperfine couplings (HFCs), again with 3d metal centers. Almost 25 years ago, Munzarová and Kaupp found that the core–shell spin polarization needed in many cases to get the isotropic metal HFC right requires hybrid DFAs with large EXX admixtures.^24,25^ At that time, only so-called global hybrids (GHs) with constant EXX admixture were available. Increased global EXX admixture helps to enhance the core–shell spin polarization (CSSP) but also increases the valence-shell spin polarization (VSSP). In cases where the singly occupied molecular orbital(s), SOMO(s), have significant metal–ligand antibonding character, the exaggerated VSSP leads to substantial spin contamination, i.e., spin-symmetry breaking, SSB, as measured by the ⟨*S*^2^⟩ expectation value. This distorts the spin-density distribution and leads to large errors in dipolar HFCs (the latter are less affected by CSSP than isotropic metal HFCs).^24,25^ The isotropic HFCs can also be affected detrimentally, but they also depend on other aspects, including, in particular, the description of CSSP. Notwithstanding the possible usefulness of allowing SSB in other contexts,^26,27^ the adverse effects of spin contamination in open-shell transition-metal complexes with GHs are not restricted to EPR parameters like HFCs^24,25^ and g-tensors^28^ but can also deteriorate the description of other quantities (for example, vibrational frequencies or even molecular structures).^29^ In the context of the above-mentioned 3d metal HFCs, we could recently demonstrate^30^ that LHs with position-dependent EXX admixture provide improvement, as the local mixing function (LMF) governing the position dependence typically will provide larger admixtures in the core region and smaller ones in the valence region. However, the LHs evaluated so far could not fully eliminate the “spin-polarization/spin-contamination” dilemma, as notable spin contamination remained present in critical cases with the available LMFs.^30^

The interrelation between SSB and static correlation is a complex one. A differentiated picture of this interrelation in transition-metal complexes has recently put forward by Shee et al.^31^ They argued that SSB does not always signal static correlation and multireference character, but can also reflect a variational collapse due to a lower-lying higher-spin state at the given computational level. If, for example, SSB at UHF level is removed by a hybrid functional with moderate EXX admixture like B3LYP, or with renormalized orbital-optimized MP2 methods, it has been suggested to have other origins than static correlation. Those authors also stated that delocalization errors (DEs) in DFAs tend to reduce SSB and thereby counter the effects of EXX admixture.^31^ While this is indeed true in many cases, in this work we will also discuss counterexamples to this rule of thumb, both for GHs and, more importantly, for LHs with sc-corrections.

Here, we apply novel scLHs and scRSLHs to the question of SSB and to the isotropic and dipolar metal HFCs (*A*_iso_ and *A*_dip_, respectively) for a small set of manganese complexes that have been in the focus of previous analyses.^24,25,30^ We will include [Mn(CN)_4_]^2–^ as a case dominated by CSSP, and [MnO_3_], [Mn(CN)_4_N]^−^ and [Mn(CN)_5_NO]^2–^ as systems with substantial VSSP and resulting SSB. Notably, some of the scRSLHs examined here include also additional corrections in their LMF to deal with DE in abnormal open-shell regions in space,^20^ and we will evaluate the effects of such corrections on SSB and HFCs. Notably, both the sc- and DE-corrections can modify the form of the LMF that governs position-dependent EXX admixture. Comparative graphical analyses of LMFs will thus be an important tool in this work.

## 2 Theory

### 2.1 Local Hybrid Functionals and Their Extensions to Include Strong Correlations and Range Separation

The position-dependent EXX admixture of LHs is a natural step forward from GHs in the quest to locally balance SIE and static correlations.^32^ An appropriate starting point for the present work is the LH20t functional^33^ that may be formulated as
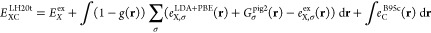
1This formulation starts from 100% exact exchange, and thus the second term may be viewed as a nonlocal correlation term^32,34^ that one may connect with nondynamical correlation (NDC), in particular with the left–right correlations in chemical bonds. The LMF *g*(**r**) defines the extent to which EXX and semilocal exchange are combined at a particular point in space. The calibration function (CF) *G*(**r**) is included to mitigate the so-called “gauge problem” of LHs, i.e., a possible mismatch between semilocal exchange and EXX energy densities, that is known to otherwise introduce artificial NDC contributions.^32^ For LH20t and most of the other LHs and RSLHs covered in the present work, the last term, which covers dynamical correlation (DC), is represented by the B95c meta-GGA.^35^

Drawing from the KP16/B13 model of strong correlation,^22^ sc-corrections have been introduced into the LH framework by using a multiplicative sc-function *q*_AC_(**r**), providing scLHs:^18^
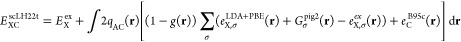
2Here, *q*_AC_(**r**) presents a local interpolation function between the KS noninteracting reference system and the fully interacting system at each point in space (local adiabatic connection, local AC). *q*_AC_(**r**) ranges from 0.5 for weakly correlated situations to 1.0 for points in space, where strong correlations dominate. The latter situations are identified by the underlying real-space function *z*(**r**), on which *q*_AC_(**r**) is built. At these points in space, kinetic-energy contributions^21,22^ to both NDC and DC correlation terms are then added via a local AC.

Different models to construct *q*_AC_(**r**) and the underlying function *z*(**r**) have been proposed for scLHs. The initial approaches^17,18^ closely followed the KP16/B13 model,^22^ where *z*(**r**) is obtained by a ratio of exchange-hole normalization constants within the reverse Becke-Roussel (revBR) machinery, with some modifications. This machinery has been introduced initially with Becke’s B05 and B13 coordinate-space models.^21,36^ Applying this construction to the LH20t functional (see above) gave the scLH22ta and scLH22t functionals.^18^ The latter of these functionals applies a damping factor for small values of *z*(**r**), which reduces double counting of NDC for weakly correlated situations and allows the parameters of LH20t to be retained without change. In contrast, scLH22ta does not involve damping and therefore provides larger corrections, with the downside that some deterioration for weakly correlated situations may occur.

A subsequent simplification^19^ of the sc-corrections based on simple ratios between semilocal and EXX energy densities avoids the complicated revBR machinery and has provided the recent scLH23t-mBR and scLH23t-mBR-P functionals. Here, *z*(**r**) is constructed from a ratio between a modified BR energy density and the EXX one, and different local AC interpolation formulas are used to obtain *q*_AC_(**r**).^19^

### 2.2 Extension to Range-Separated Local Hybrids: Introduction of a DE Correction

Since LHs, like LH20t, are principally unable to give the correct asymptotic potential at large distance from the nuclei in a system,^37^ we have recently developed and implemented the ωLH22t range-separated LH (RSLH),^38^ which can be written as
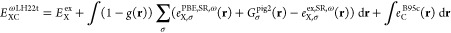
3Since ωLH22t exhibits full long-range (LR) EXX admixture, it provides excellent performance, e.g., in TDDFT calculations of excitations with charge-transfer character. ωLH22t is also one of the best-performing rung 4 functionals for a wide variety of other data, including the GMTKN55 main-group test suite and several organometallic transition-metal reaction energy and barrier benchmarks.^38^ Most notably, ωLH22t provides excellent frontier-orbital energies for ionization potentials, electron affinities and fundamental band gaps for wide variety of relevant systems in molecular electronics and organic photovoltaics.^39^ In this context, ωLH22t reduces significantly the DEs and FCEs of the underlying LH20t functional. However, the long-range EXX admixture also increases FSEs and thus is not very well-suited for systems with substantial static correlations.

Based on ωLH22t, we have therefore recently introduced scRSLHs that dramatically reduce FSEs and improve spin-restricted bond dissociation curves.^20^ The middle nonlocal correlation term in eq 3 is based exclusively on short-range (SR) exchange-energy densities. A straightforward application of a function *q*_AC_(**r**) to the NDC and DC contributions is therefore not fully effective in introducing strong correlations, which are, in essence, LR in nature. The formulation for scRSLHs (eq 4) therefore additionally introduces an extra LR contribution governed by a switching function *f*_FR_(**r**) (where FR denotes the introduction of full-range EXX). This function depends in a similar way on real-space function *z*(**r**) and on the same adjustable parameters as *q*_AC_(**r**). However, while *q*_AC_(**r**) ranges from 0.5 to 1.0, *f*_FR_(**r**) ranges from 0.0 for weak correlations to 1.0 for a dominance of strong correlations.^20^ This construction indeed reduces dramatically FSEs. Due to the inclusion of damping factors in *q*_AC_(**r**) and *f*_FR_(**r**), the correct LR asymptotic potential is largely retained in weakly correlated situations.^20^
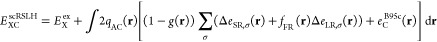
4with
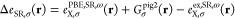
5and
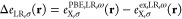
6

Different constructions for *q*_AC_(**r**) and *f*_FR_(**r**) have provided the functionals ωLH23tB, ωLH23tE, and ωLH23tP.^20^ ωLH23tB is analogous to scLH22t as it is still based on the revBR machinery from KP16/B13. ωLH23tE and ωLH23tP are constructed more analogously to scLH23t-mBR and scLH23t-mBR-P, respectively, by using a simple ratio between semilocal (mBR) and EXX energy densities. They differ in the form of the local AC interpolation: while ωLH23tE uses a simple error function, ωLH23tP uses a more flexible Padé function to reduce local maxima in spin-restricted dimer dissociation curves.^20^

As the present work concentrates on spin-polarized open-shell systems, the recently introduced additional DE correction (DEC) to the LMF^20^ becomes relevant. It was found that the performance of ωLH22t and the derived scRSLHs for specific open-shell systems could be improved by enhancing EXX admixture locally by an additional DEC term in the LMF, that is also constructed from *z*(**r**).^20^ In contrast to the sc-corrections, it aims to reduce the DE locally, in analogy to the second part of the LMF of the PSTS functional.^40^ The resulting modified LMF can be written as
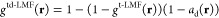
7with
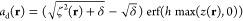
8where ζ is the spin polarization, δ = 0.00001, and *h* is an adjustable parameter.^20^

## 3 Computational Details

Most calculations were performed using a prerelease version of Turbomole 7.8,^41,42^ in which the functionals discussed above have been implemented. The two-electron integrals necessary for range-separated and full exact-exchange energy densities were calculated through seminumerical integration techniques, as discussed previously,^38,43−46^ using the standard screening settings of Turbomole.

The main focus of this work is on several scLHs and scRSLHs: scLH21ct-SVWN-m^17^ is most closely related to the simple first-generation LDA-based LHs LH12ct-SsifPW92 and LH12ct-SsirPW92,^47^ which are also included. scLH22t and scLH22ta^18^ are based on LH20t.^33^ So are scLH23t-mBR and scLH23t-mBR-P,^19^ for which results will mostly be reported as part of the Supporting Information. Starting from the RSLH ωLH22t,^38^ three scRSLHs without DE-correction terms (ωLH23tX, X = B, E, P)^20^ will be evaluated. Adding the DE corrections to ωLH22t gives ωLH23td, and adding them to the three scRSLHs provides the DE-corrected scRSLHs ωLH23tdX (X = B, E, P).^20^ Further results are provided for LH23pt^48^ to probe the effect of a modified core LMF for isotropic HFCs, and for the KP16/B13 functional.^22^ As reference examples for a GGA functional and for a GH with medium EXX admixture, we have chosen PBE^49^ and PBE0,^50^ respectively. These have been studied before for these complexes, together with many more functionals.^24,25,30^ The exchange-correlation functionals evaluated in this work are collected in Table 1.

**Table 1:** Exchange-Correlation Functionals Evaluated in This Work

	type	parent LH	DEC	*q*_AC_	ref
PBE	GGA	–	–	–	(49)
PBE0	GH	–	–	–	(50)
					
LH12ct-SsifPW92	LH	–	–	–	(47)
LH12ct-SsirPW92	LH	–	–	–	(47)
LH20t	LH	–	–	–	(33)
ωLH22t	RSLH	–	–	–	(38)
LH23pt^a^	LH	–	–	–	(48)
					
scLH21ct-SVWN-m	LH	–b^,^^c^	–	mKP16^d^	(17)
scLH22t	LH	LH20t	–	mKP16	(18)
scLH22ta	LH	LH20t^c^	–	mKP16^d^	(18)
scLH23t-mBR	LH	LH20t	–	erf	(19)
scLH23t-mBR-P	LH	LH20t	–	Padé	(19)
					
ωLH23tE	RSLH	ωLH22t	–	erf	(20)
ωLH23tB	RSLH	ωLH22t	–	mKP16	(20)
ωLH23tP	RSLH	ωLH22t	–	Padé	(20)
					
ωLH23td	RSLH	ωLH22t	+	–	(20)
					
ωLH23tdE	RSLH	ωLH22t	+	erf	(20)
ωLH23tdB	RSLH	ωLH22t	+	mKP16	(20)
ωLH23tdP	RSLH	ωLH22t	+	Padé	(20)

a A pt-LMF optimized to improve core properties is used for LH23pt, instead of a t-LMF.

b This scLH is most closely related to the LH12ct-SsifPW92 and LH12ct-SsirPW92 functionals.

c Parameters of the underlying LH have been reoptimized in the presence of *q*_AC_.

d Undamped version of *q*_AC_^mKP16^.

Unless specified otherwise, calculations of HFCs used the scalar relativistic X2C Hamiltonian^51,52^ and the corresponding picture-change corrected HFC operator,^53^ even though relativistic effects for these Mn complexes are known to be small.^30^ These calculations employed the fully uncontracted versions of the NMR_9s7p4d^24^ and IGLO-III^54^ basis sets to maintain consistency with previous work. Additionally, Turbomole’s “universal” auxiliary basis sets^55^ were used for the resolution of identity (RI-J) approach to the Coulomb integrals.^56,57^ Turbomole gridsize 3 was used throughout, the self-consistent field (SCF) convergence criterion was set to 10^–8^ a.u.

Separate nonrelativistic calculations of ⟨*S*^2^⟩ and *A*_iso_ with the DM21^58^ functional were done using its implementation interfaced to the PySCF 2.0 program.^59,60^ DM21 is a completely black-box deep-neural network functional exhibiting tens of thousands of parameters. It has been described as an RSLH^58^ and has been trained with extensive FCE and FSE data. DM21 indeed provides very small FSEs. It is thus of interest to probe how DM21 deals with the SSB in the present complexes. We were unable to extract complete and meaningful HFC data directly from the PySCF code, but we have extracted the spin density at the Mn nucleus and then converted it to the isotropic HFC. These calculations used the same basis sets as described above, a full calculation of the Coulomb contribution (without RI), PySCF grid size 3, and an SCF energy convergence criterion of 10^–7^ a.u. In the case of [Mn(CN)_5_NO]^2–^, these computations were restricted to the isolated dianion (BP86-optimized structure), in contrast to the full embedded-cluster treatment described below.

The experimental solid-state structure^61^ was used for [Mn(CN)_4_]^2–^, as in previous work.^24,30^ Also for consistency with those previous studies, B3LYP-optimized structures were used for MnO_3_ and [Mn(CN)_4_N]^−^. Additional all-electron structure optimizations used the BP86^62,63^ functional to assess the effect of having less SSB during optimization, or again B3LYP.^64,65^ These nonrelativistic optimizations used def2-TZVP^66^ basis sets and corresponding auxiliary basis sets for the RI-J method. Weight derivatives and grid size 3 were used, together with energy and gradient convergence thresholds of 10^–7^ a.u. and 10^–4^ a.u., respectively.

Initial modeling of environmental effects used the COSMO^67−70^ model, with dielectric constant ε = 8.9 for [Mn(CN)_4_]^2–^ to simulate CH_2_Cl_2_, and with ε = 37.5 for [Mn(CN)_5_N]^−^ to simulate acetonitrile. ε = 4.0, ε = 78.4, and ε = *∞* were used to explore various environmental conditions in an initial effort for [Mn(CN)_5_NO]^2–^. These evaluations can be found in the Supporting Information.

Given the 2-fold negative charge of [Mn(CN)_5_NO]^2–^ and the fact that the experimental EPR data were obtained for the complex doped into a single crystal of the diamagnetic host lattice of the Na_2_Fe(CN)_5_NO·2H_2_O,^71^ more detailed environmental modeling was undertaken for this system. We optimized the structure of the Na_2_Fe(CN)_5_NO·2H_2_O solid using periodic boundary conditions in the riper module^72,73^ of Turbomole at the BP86/pob-TZVP^74^ level, using D3 dispersion corrections^75^ with Becke-Johnson damping.^76^ All calculations employed the settings previously mentioned, as well as grid size 5, 2 *k*-points along the shortest cell dimensions and an SCF convergence criterion of 10^–9^ a.u. Initial coordinates and the cell parameters were taken from ref (77). The initial positions of the hydrogen atoms of the water molecules were chosen by chemical intuition while respecting the full *P_nnm_* space group symmetry of the lattice. The unit-cell dimensions were kept constant throughout the calculation. A [Na_16_(Fe(CN)_5_NO)_2_·8H_2_O]^12+^ cluster containing two Fe-centers and having full C_2*h*_ point-group symmetry of the cell was cut from the converged periodic structure. The final structure was obtained by replacing one Fe center in the cluster model by Mn and relaxing the metal–ligand distances at the r^2^SCAN-3c^78^ composite level or using the BP86 functional.

We note that the ⟨*S*^2^⟩ expectation value used is that of the KS noninteracting reference determinant and not the proper value for the interacting system. Previous experience suggests that this, nevertheless, does provide a good measure of the detrimental effects of SSB, e.g., regarding the dipolar HFC.^24,30^ Similar views of the usefulness of ⟨*S*^2^⟩ taken from the KS-determinant have been expressed in other contexts.^31,79^

## 4 Results and Discussion

We will look at four different Mn complexes to cover various situations. [Mn(CN)_4_]^2–^ is included as an example that does not exhibit large VSSP nor SSB, and can be used to evaluate the suitability of the various functionals for treating the CSSP. The complexity of the description is then increased stepwise, starting from MnO_3_, which clearly exhibits SSB but does not require any modeling of the environment. [Mn(CN)_4_N]^−^ also exhibits notable VSSP, and a continuum solvent model is sufficient to describe the environment realistically. The most demanding and difficult case is provided by [Mn(CN)_5_NO]^2–^, where the magnitude of SSB depends crucially on a detailed modeling of the environment.

Our main focus will be on SSB, with the key metrics of the ⟨*S*^2^⟩ expectation value and of the dipolar HFC, *A*_dip_. The latter can reflect SSB very directly via the distorted spin-density distribution. We will evaluate the effects of both the sc- and DE-corrections on these quantities. We will furthermore use three-dimensional (3D) plots of spin-density distributions and one-dimensional (1D) LMF plots to obtain further insights. The focus in the main text will be only on selected functionals, while additional data are provided in the Supporting Information.

Subsequently, we will look at the isotropic HFC. While *A*_iso_ is also affected indirectly by SSB, it tends to depend often crucially also on CSSP, the description of which tends to be improved by EXX admixture. In this context, we will analyze orbital contributions to *A*_iso_, in particular the metal 3s/2s ratio of CSSP contributions, as this has been found to be an important probe of the balance of CSSP.^25,30^

### 4.1 Spin Symmetry Breaking and Dipolar HFCs

#### 4.1.1 [Mn(CN)_4_]^2–^, a Pure CSSP Case

Since all five singly unoccupied MOs (SOMOs) of ^6^[Mn(CN)_4_]^2–^ are essentially pure metal d-orbitals, VSSP and thus SSB are minimal for this complex, while CSSP of the metal 2s/3s shells is crucial for the negative *A*_iso_. This system should thus serve well as a reference system, where we want to see if the sc-corrected functionals preserve the performance of the underlying LH20t or ωLH22t functionals, respectively. For the other complexes, we will separate the discussion of VSSP and SSB/*A*_dip_ from that of CCSP and *A*_iso_. Here we can state directly that the sc-corrected functionals do not alter the overall results of the underlying LHs or RSLH. Table S1 in the Supporting Information summarizes ⟨*S*^2^⟩, as well as *A*_iso_ and its orbital contributions. The ^55^Mn *A*_dip_ vanishes due to the tetrahedral symmetry. As expected, SSB is vanishingly small here for all functionals, irrespective of sc- or DE-corrections. *A*_iso_ values also do not change very much, compared to the underlying LH20t, LH12ct-SsirPW92, or ωLH22t reference points, respectively, except for minor reductions by a few MHz for the “undamped” scLHs and for the DE-corrected ωLH23td and its three sc-corrected variants. The simpler LDA-based LHs and the related scLH21ct-SVWN-m give noticeably more negative *A*_iso_ values, closer to the −199 MHz experimental value in solution, in line with previous results.^30^ These outcomes also do not change significantly when replacing the experimental condensed-phase stucture by optimized structures at BP86 or B3LYP levels or when including a COSMO solvent model in the HFC computations (see Table S2 in the Supporting Information). A modification of the core LMF, compared to LH20t, is provided by LH23pt, and it also gives somewhat more negative *A*_iso_ values. While the isotropic HFCs are not the central goals of this work, the optimal treatment of CSSP in such metal complexes clearly warrants further investigation. We have also included here results for PBE as an example of a GGA functional and PBE0 as a GH (with 25% EXX admixture). As found previously,^24,30^ GGAs substantially underestimate the CSSP and, thus, give too small negative contributions to *A*_iso_. PBE0 enhances the CSSP to the extent where *A*_iso_ is comparable to the LH20t or ωLH22t results (falling short of the LH12ct-type functionals or experiment). Notably, we will see below that a 25% constant EXX admixture leads already to substantial SSB in VSSP cases.

#### 4.1.2 MnO_3_, a Significant VSSP Case

Based on earlier results,^24,30^ MnO_3_ has been chosen as a comparably small complex with only small environmental effects (the experimental EPR data have been obtained in Ne matrix at 4 K^80^) but with significant VSSP and thus potentially SSB. Table 2 shows computed data with various functionals for HFCs and ⟨*S*^2^⟩ expectation values. As found previously, LH20t gives substantial SSB, comparable to GHs with moderate EXX admixture like B3LYP, somewhat less than PBE0, significantly less than GHs with EXX admixtures ≥40% needed to describe CSSP correctly (as seen in the absence of notable VSSP, e.g., for [Mn(CN)_4_]^2–^, see above). Consequently, the experimental *A*_dip_ is overestimated strongly. Interestingly, the long-range EXX admixture in the ωLH22t RSLH does not change results much from LH20t, with similar SSB and a similar overestimation of *A*_dip_. We also note that the LH20t results and those with the older LH12ct-SsifPW92 and LH12ct-SsirPW92 functionals are similar.

**Table 2:** Comparison of Different Functionals, Including Those Having sc- and DE-Corrections, for ^55^Mn HFCs (in MHz; Including the 3s/2s Ratio of CSSP Contributions to *A*_iso_) and SSB of MnO_3_^a^

			*A*_iso_			
	DEC	*q*_AC_(**r**)	3s/2s	total	*A*_dip_	⟨*S*^2^⟩
PBE	**–**	**–**	–0.51	1825.0	94.6	0.771
PBE0	**–**	**–**	–0.58	1427.7	137.8	1.050
						
LH12ct-SsifPW92	**–**	**–**	–0.48	1469.4	131.8	0.931
LH12ct-SsirPW92	**–**	**–**	–0.48	1511.9	127.4	0.896
LH20t	**–**	**–**	–0.58	1504.5	130.5	0.924
ωLH22t	**–**	**–**	–0.63	1523.5	133.8	0.926
LH23pt	**–**	**–**	–0.57	1454.7	131.6	0.954
						
scLH21ct-SVWN-m	**–**	**+**	–0.11	1768.1	96.4	0.761
scLH22t	**–**	**+**	–0.58	1612.9	102.1	0.772
scLH22ta	**–**	**+**	–0.48	1733.4	95.0	0.763
						
ωLH23tE	**–**	**+**	–0.64	1542.4	117.0	0.825
ωLH23tB	**–**	**+**	–0.64	1635.9	103.0	0.770
ωLH23tP	**–**	**+**	–0.64	1541.1	117.5	0.828
						
ωLH23td	**+**	**–**	–0.23	1444.5	97.9	0.755
						
ωLH23tdE	**+**	**+**	–0.21	1424.3	96.7	0.754
ωLH23tdB	**+**	**+**	–0.16	1410.1	93.4	0.755
ωLH23tdP	**+**	**+**	–0.11	1408.1	95.8	0.754
						
Exp^b^				1613	81	0.750

a For more details on the CSSP contributions, see Table S3 in the Supporting Information.

b Data taken from ref (80).

We turn to the functionals with sc- and DE-corrections. The scLHs scLH22t and scLH22ta reduce SSB compared to the underlying LH20t, with a concomitant reduction in the overestimated *A*_dip_. As one might expect, scLH22ta, which lacks damping in the sc-factor, provides somewhat larger corrections. The simpler LDA-based, also undamped, scLH21ct-SVWN-m reduces SSB and the overestimation of *A*_dip_ to a similar extent as scLH22ta.

The scRSLH models ωLH23tX (X = E, B, P) without DE-correction perform somewhat similar as scLH22t, by reducing SSB and *A*_dip_ somewhat less than the undamped scLHs, in this case, in comparison with the underlying ωLH22t. The most striking observation is that the ωLH23td RSLH, which has a DE- but no sc-correction, reduces SSB and *A*_dip_ to a similar extent as the undamped scLHs. This performance then is retained upon adding the sc-corrections in the ωLH23tdX (X = E, B, P) DFAs. Both sc- and DE-corrections are thus able to reduce SSB and the resulting overestimation of *A*_dip_ in MnO_3_ to similar extents. We note, in passing, that using different input structures does not alter the results much (see Table S4 in the Supporting Information).

**Figure 1:**
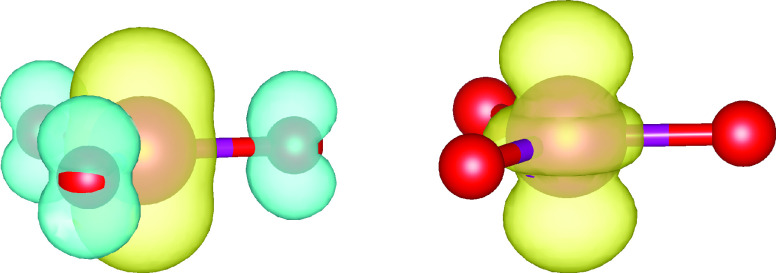
Spin-density isosurface plot (±0.011 a.u.) for MnO_3_ obtained with ωLH22t (left) and ωLH23td (right).

Let us analyze these results for MnO_3_ in more detail, focusing on a comparison of the RSLH and scRSLH models. In this case, adding just the sc-corrections in ωLH23tE or ωLH23tP provided insufficient corrections to remove SSB completely. We focus on ωLH23tB, which provides larger corrections, in comparison with the underlying ωLH22t RSLH. We will additionally look at ωLH23td, which adds only the DEC terms but not the sc-corrections. The SSB is related to exaggerated VSSP and the ensuing negative spin density. Figure 1 compares spin-density isosurface plots for ωLH22t and its DE-corrected ωLH23td version (see Figure S1 for other functionals). The large negative spin density on the oxygen atoms, balanced by enhanced positive spin density in the Mn valence shell, is responsible for the spin contamination with the uncorrected ωLH22t. It is effectively removed by the DE-correction. This can be traced largely to just two valence MOs, MOs 19 and 20, as shown by the Mulliken d-orbital populations for both spin channels in Table 3 (also see Figures S2 and S3). The spin polarization of these two MOs is substantially reduced by the DE-correction (Table 3). It occurs now exclusively in the metal d-orbitals, removing the negative spin density on oxygen (cf. Figures S2 and S3). Introducing just the sc-corrections, as for example with ωLH23tB, also reduces and localizes the spin polarization of MOs 19 and 20 (Table 3), but to a lesser extent. Adding both DE- and sc-corrections in ωLH23tdB has the overall largest effect, i.e., the sc-correction helps to further reduce VSSP and thus SSB (Figure S1).

**Table 3:** Effects of sc- and DE-Corrections on the Main Mulliken Spin-Population Contributions to Spin Polarization in MnO_3_

		d-contribution		
	MO	alpha	beta	type of AO
ωLH22t	19	0.620	0.342	d_*yz*_
	20	0.620	0.342	d_*xz*_
				
ωLH23td	19	0.491	0.466	d_*yz*_
	20	0.491	0.466	d_*xz*_
				
ωLH23tB	19	0.525	0.441	d_*yz*_
	20	0.525	0.441	d_*xz*_
				
ωLH23tdB	19	0.492	0.476	d_*yz*_
	20	0.492	0.476	d_*xz*_

**Figure 2:**
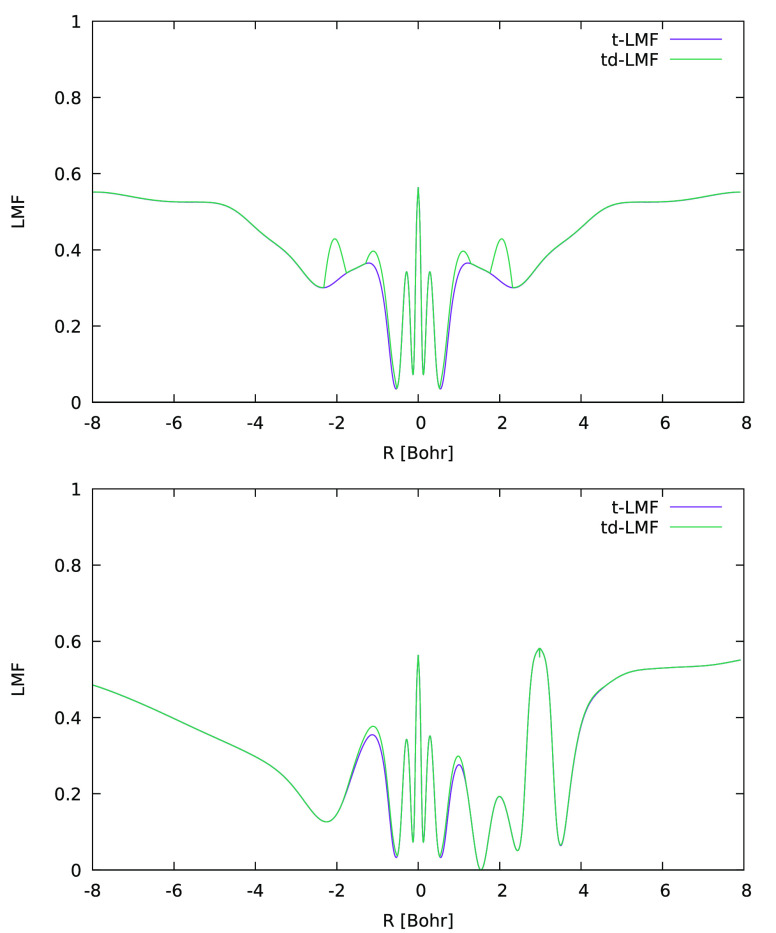
One-dimensional (1D) graphical comparison of t-LMF (ωLH22t) and DE-corrected td-LMF (ωLH23td) for MnO_3_ along lines passing through the Mn atom, perpendicular to the molecular plane (top) and along an Mn–O bond (bottom).

These results seem contradictory at first sight, as we know the sc-corrections locally reduce the EXX admixture^18^ while the DE-corrections locally enhance the EXX admixture.^20^ How could both corrections help reduce VSSP and thus SSB? Since MnO_3_ is a comparably small and “simple” molecule, it seems well-suited for further analyses. We therefore plot LMFs with and without sc- and DE-corrections at different positions and in different directions. Figure 2 shows such plots for the effect of the DE-corrections along lines passing through the Mn nucleus. Obviously, the EXX admixture is enhanced in the Mn valence shell, more so perpendicular to the molecular plane than along the Mn–O bond. This occurs in a region of space dominated by the Mn valence shell, where the metal d-orbitals have large amplitude. Very little change is seen around the oxygen atoms. Why should a local enhancement of EXX admixture near the metal center by the DE-corrections diminish the negative spin density on the oxygen atoms? Some years ago, Remenyi and Kaupp had analyzed spin-density distributions and EPR parameters for a series of ruthenium complexes with redox-noninnocent ligands, comparing BP86 results with GHs (B3LYP, BHLYP) featuring increasing (global) EXX admixtures.^81^ Interestingly, they found for a number of cationic complexes with predominantly metal-centered spin density that SSB increased from BP86 to B3LYP but then decreased to BHLYP (as measured by ⟨*S*^2^⟩ expectation values). This unexpected nonlinear trend has been rationalized as follows:^81^ from the BP86 GGA functional to the B3LYP GH with 20% EXX admixture, the spin polarization on the most strongly bound ligand atoms was increased, leading to the larger SSB. When moving to 50% EXX admixture with BHLYP, the spin delocalization from the open-shell metal center to the relevant ligand atoms was largely surpressed by smaller DEs. Therefore, simply not much SOMO spin density was left in the vicinity of those ligand atoms, which could polarize the doubly occupied orbitals on the ligand. The DE-corrections operate in a similar way, by enhancing locally EXX admixture in the metal valence shell, thereby reducing the general spin-density delocalization onto the relevant ligand atoms. More specifically, for MnO_3_ the delocalization of spin density from the metal d_*xz*_ and d_*yz*_ orbitals into the oxygen p_*z*_ orbitals is reduced. We note that the nonlinear trend with EXX admixture for cationic Ru complexes with quinoid ligands in ref (81) occurred only with N/O or O/O combinations of the ligating atoms, while for N/S and S/S bonding patterns, SSB increased further from B3LYP to BHLYP. In the latter cases, the M-S antibonding nature of the SOMO remained large even with a 50% EXX admixture, and then the VSSP and the resulting SSB was actually enhanced. In contrast, with N/O and O/O ligating atoms, the bond ionicity was larger, and then with a 50% EXX admixture, the SOMO was mostly nonbonding, leading to lower VSSP and lower SSB with BHLYP.^81^ All three VSSP cases studied here have at least one strong covalent interaction, and thus a larger constant EXX admixture will inevitably increase SSB. Nevertheless, such covalency aspects also come into play regarding the effects of the DE-corrections.

**Figure 3:**
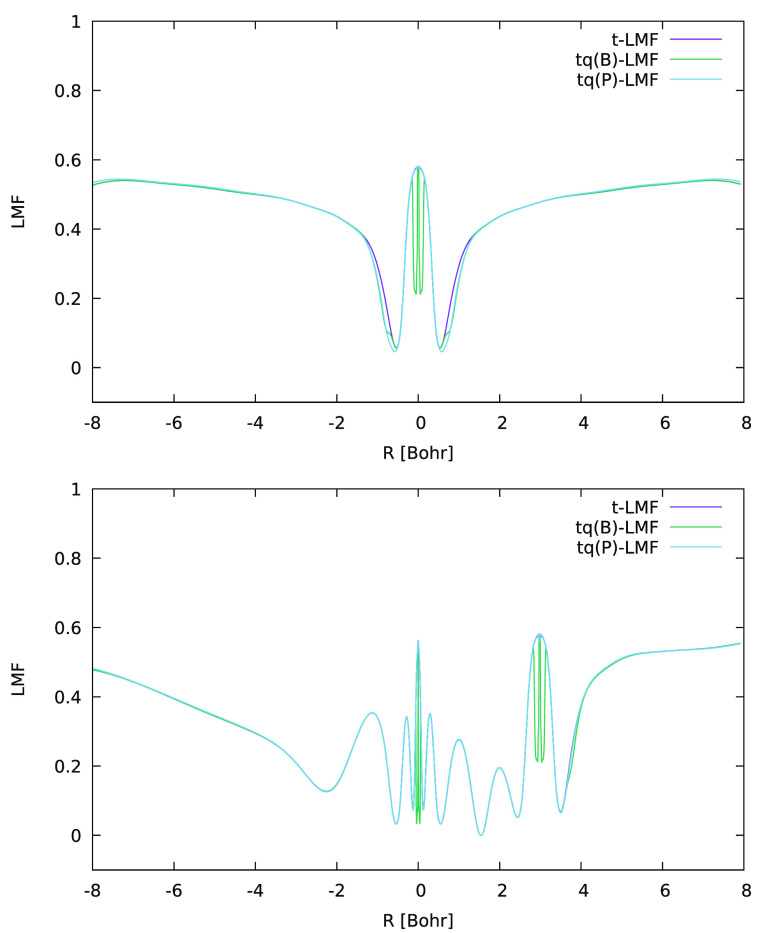
One-dimensional (1D) graphical comparison of t-LMF (ωLH22t) and sc-corrected tq-LMF (ωLH23tB and ωLH23tP) for MnO_3_ along lines passing through an oxygen atom, perpendicular to the molecular plane (top) and along an Mn–O bond (bottom).

The sc-corrections operate differently: as shown in Figure 3 for ωLH23tB and ωLH23tP, the sc-corrected LMFs somewhat decrease the EXX admixture near the oxygen atoms, particularly perpendicular to the bonds, while having almost no effect near the metal center. This appears to diminish directly spin polarization at those very oxygen atoms. Alterations near the nucleus for ωLH23tB are artifacts arising from the numerically difficult revBR machinery.^19^ They are absent for ωLH23tP, which is based on simpler measures (see the Theory section). On the other hand, ωLH23tP reduces SSB less effectively than ωLH23tB. Why this is the case is currently unclear. It is interesting to note, however, that the sc-factors in ωLH23tP have been optimized to minimize unphysical local maxima in spin-restricted bond dissociation curves, which may be related to intermediate-strength static correlation aspects. Finally, we can state that adding both sc- and DE-corrections, as in ωLH23tdB, adds both above-mentioned changes to the LMF, i.e., a local enhancement of EXX admixture in the metal valence shell and a local decrease in the oxygen valence shell (not shown).

#### 4.1.3 [Mn(CN)_4_N]^−^, a Different VSSP Case

This monoanionic nitrido complex has also been identified as a system exhibiting substantial SSB with hybrid functionals, comparable in magnitude with MnO_3_.^24,25,30^ Yet, the electronic structure is notably different, as VSSP and SSB arise largely from the strong multiply bound nitrido ligand and the associated π-antibonding nature of the SOMO.^25^ LH20t and ωLH22t again provide ⟨*S*^2^⟩ values of ∼0.9 for this doublet complex. The results in Table 4 are from gas-phase computations, while the experimental EPR data have been obtained in acetonitrile liquid and frozen solution.^82^ However, adding a COSMO solvent model for acetonitrile changes the data very little, as do different starting structures (see Table S6 in the Supporting Information).

**Table 4:** Comparison of Different Functionals, Including Those Having sc- and DE-Corrections, for ^55^Mn HFCs (in MHz; Including the 3s/2s ratio of CSSP Contributions to *A*_iso_^a^) and SSB of [Mn(CN)_4_N]^−^

			*A*_iso_			
	DEC	*q*_AC_(**r**)	3s/2s	total	*A*_dip_	⟨*S*^2^⟩
PBE	**–**	**–**	–0.50	–156.8	–115.1	0.774
PBE0	**–**	**–**	–0.51	–286.0	–111.4	0.981
						
LH12ct-SsifPW92	**–**	**–**	–0.48	–330.0	–116.8	0.903
LH12ct-SsirPW92	**–**	**–**	–0.48	–308.9	–117.8	0.880
LH20t	**–**	**–**	–0.55	–252.0	–115.6	0.903
ωLH22t	**–**	**–**	–0.56	–265.6	–117.0	0.933
LH23pt	**–**	**–**	–0.51	–289.3	–114.4	0.928
						
scLH21ct-SVWN-m	**–**	**+**	–0.34	–201.9	–124.1	0.768
scLH22t	**–**	**+**	–0.53	–182.6	–128.6	0.792
scLH22ta	**–**	**+**	–0.51	–118.4	–124.0	0.774
						
ωLH23tE	**–**	**+**	–0.56	–219.1	–126.0	0.827
ωLH23tB	**–**	**+**	–0.55	–173.2	–134.3	0.783
ωLH23tP	**–**	**+**	–0.56	–179.8	–130.7	0.784
						
ωLH23td	**+**	**–**	–0.17	–299.7	–139.3	0.756
						
ωLH23tdE	**+**	**+**	–0.16	–295.1	–138.8	0.755
ωLH23tdB	**+**	**+**	–0.13	–268.2	–139.4	0.754
ωLH23tdP	**+**	**+**	–0.06	–295.8	–137.4	0.754
						
Exp^b^				–276	–122.4	0.750

a For more details on the CSSP contributions, see Table S5 in the Supporting Information.

b Data have been taken from ref (82).

We start with the scLHs. As discussed above for MnO_3_, the sc-corrections reduce SSB, with the undamped models giving the larger changes. The correlation with *A*_dip_ is not as clear as for the other two VSSP cases studied here, although differences are, in fact, small. For example, scLH22t leads to ∼4 MHz larger changes from LH20t than the undamped scLH22ta or scLH21ct-SVWN-m (Table 4). This is consistent with observations for GHs in the original study,^24^ where moderate SSB with 20% EXX admixture changed *A*_dip_ only slightly, compared to GGAs (cf. the slightly more negative *A*_dip_ for PBE vs PBE0 in Table 4, where one would expect a larger effect for the GH).

For the ωLH23tX scRSLHs, the *A*_dip_ values also do not vary very much. Adding the DE-corrections eliminates SSB almost completely, even without the sc-corrections (ωLH23td), which shifts *A*_dip_ slightly more than just the sc-corrections. We do not attach too much significance to the fact that the experimental *A*_dip_ value is overshot somewhat upon eliminating SSB, as effects of an incomplete treatment of environmental effects by a dielectric continuum model might still well be on the order of magnitude of these changes.

Figure S4 shows that, analogous to the negative spin density on oxygen in MnO_3_ (see above), the SSB arises from negative spin density on the nitrido ligand, accompanied by an enhanced positive spin density at the metal center. DE corrections are more effective in reducing these SSB artifacts than sc-corrections, while undamped scLHs are more effective than damped scLHs, or (also damped) scRSLHs without DE-term. DE-corrections again increase the local EXX admixture in the metal valence shell (see Figure S5). In this case, the undamped sc-corrections of scLH22ta reduce the local EXX admixture notably both on the nitiride ligand and in the metal valence shell, mostly on the opposite side to the nitride ligand (see Figure S6). The effects for scLH22t or the scRSLHs without DE-corrections are smaller but qualitatively similar.

#### 4.1.4 [Mn(CN)_5_NO]^2–^, an Extreme VSSP Case

The free [Mn(CN)_5_NO]^2–^ dianion has been found to exhibit extreme SSB.^24,30^ For GHs with a moderate EXX admixture like B3LYP, ⟨*S*^2^⟩ values of ∼1.4 have been found for this doublet dianion. LH20t or ωLH22t give similar values of ∼1.3–1.4 (Table S8 in the Supporting Information). Embedding in a COSMO solvent model with dielectric constant ϵ = 4.0 or ϵ = 78.4 reduces these values, but not by much (see Table S8). Consequently, the negative *A*_dip_ is underestimated in absolute value by almost a factor 2. Use of BP86-optimized structures and/or COSMO embedding further reduce the SSB somewhat, and we provide further data in the Supporting Information (Tables S8 and S9).

The experimental HFCs have been measured for the complex doped into a single crystal of the diamagnetic host lattice of Na_2_Fe(CN)_5_NO·2H_2_O.^71^ We therefore decided to focus the following analyses on a more realistic embedded-cluster model for [Mn(CN)_5_NO]^2–^ (see the Computational Details section). The stabilization of the negative charge by the counterions in the host crystal, in fact, reduces SSB to an extent, where ⟨*S*^2^⟩ is only moderately larger than the values discussed above for MnO_3_ or [Mn(CN)_4_N]^−^ (Table 5). This leaves this system nevertheless the most pronounced VSSP/SSB case studied in this work. Note that the cluster embedding also affects both the computed *A*_dip_ and *A*_iso_ values to the extent that the results for the best-performing functionals become better aligned with the experiment (see below).

**Table 5:** Comparison of Different Functionals, Including Those Having sc- and DE-Corrections for ^55^Mn HFCs (in MHz; Including the 3s/2s Ratio of CSSP Contributions to *A*_iso_^a^) and SSB of [Mn(CN)_5_NO]^2–^ with Cluster Embedding,^a^^,^^b^ Using a BP86 Optimized Structure

			*A*_iso_			
	DEC	*q*_Ac_(**r**)	3s/2s	total	*A*_dip_	⟨*S*^2^⟩
PBE	**–**	**–**	–0.46	–104.6	–100.3	0.779
PBE0	**–**	**–**	–0.50	–191.2	–79.3	1.035
						
LH12ct-Ssifpw92	**–**	**–**	–0.46	–233.8	–88.3	0.935
LH12ct-Ssirpw92	**–**	**–**	–0.46	–219.6	–90.5	0.912
LH20t	**–**	**–**	–0.53	–160.4	–87.0	0.952
ΩLH22t	**–**	**–**	–0.55	–170.3	–83.8	1.016
LH23pt	**–**	**–**	–0.51	–188.6	–84.7	0.972
						
sLH21ct-SVWN-m	**+**	**–**	–0.34	–153.9	–109.2	0.770
scLH22t	**+**	**–**	–0.53	–151.1	–92.8	0.898
scLH22ta	**+**	**–**	–0.51	–68.9	–108.2	0.778
						
ωLH23tE	**+**	**–**	–0.55	–170.4	–84.3	1.011
ωLH23tB	**+**	**–**	–0.54	–161.7	–88.7	0.962
ωLH23tP	**+**	**–**	–0.55	–169.3	–85.2	1.000
						
ωLH22td	**–**	**+**	–0.11	–249.1	–124.5	0.764
						
ωLH23tdE	**+**	**+**	–0.11	–248.8	–125.1	0.763
ωLH23tdB	**+**	**+**	–0.08	–231.5	–126.4	0.760
ωLH23tdP	**+**	**+**	0.00	–256.3	–125.2	0.763
						
Exp^c^				–219.5	–115.2	0.750

a For more details on the CSSP contributions, see Table S7 in the Supporting Information.

b See the Computational Details section.

c Data taken from ref (71).

The pronounced SSB with, e.g., PBE0, LH20t, or ωLH22t gives rise to insufficiently negative *A*_dip_ values. Observations for the three scLHs match the above observations for MnO_3_ and [Mn(CN)_4_N]^−^: the damped scLH22t provides only partial corrections to SSB and to *A*_dip_, the undamped scLH22ta and scLH21ct-SVWN-m give rise to larger changes. For the damped scRSLHs without DE-corrections, even for this embedded cluster model, the reduction of SSB is clearly insufficient; consequently, *A*_dip_ also changes very little, compared to ωLH22t. In contrast, introduction of the DE-correction in ωLH23td removes most of the SSB and provides substantial changes to *A*_dip_ (overshooting slightly). This is then retained upon adding sc-corrections in the ωLH23tdX scRSLHs. We note in passing that for the isolated dianion or for COSMO embedding, the DE-correction alone is not sufficient to correct SSB fundamentally, and only the combined use of DE- and sc-corrections affects this change (see Tables S8 and S9).

**Figure 4:**
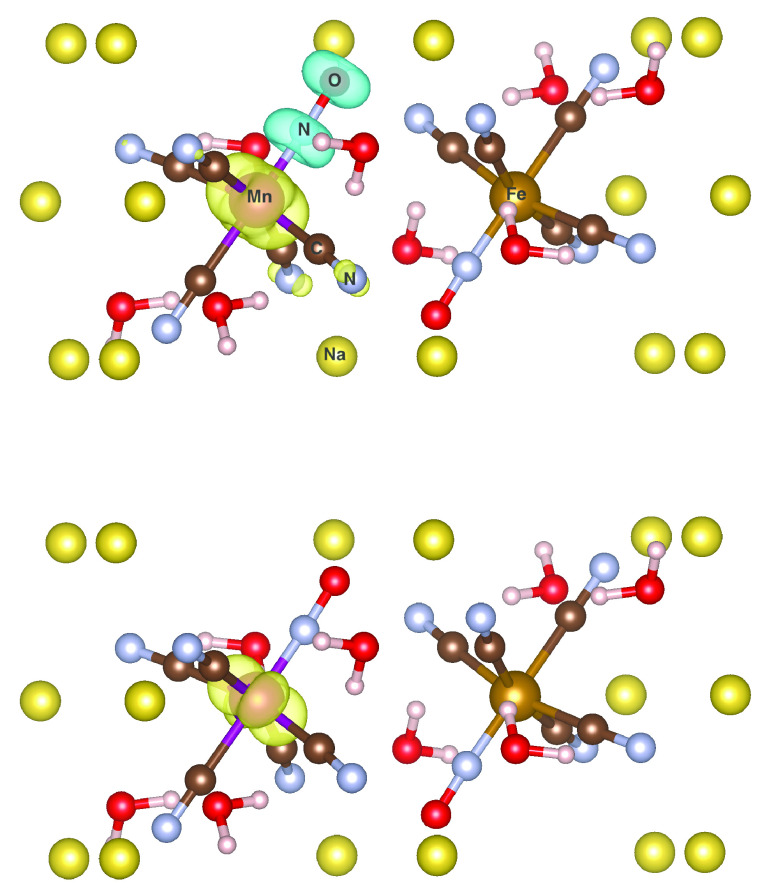
Spin-density isosurface plot (±0.011 a.u.) for cluster-embedded [Mn(CN)_5_NO]^2–^, comparing ωLH22t (top) and ωLH23td (bottom).

For closer analysis of SSB, Figure 4 provides a comparison of spin densities for the embedded cluster, comparing ωLH22t with ωLH23td (see Figure S8 for other functionals). Analogous to the other VSSP cases above, here, it is, in particular, a negative spin density on the NO ligand, accompanied by enhanced spin density in the metal valence shell, that is responsible for the SSB with the uncorrected RSLH. The SSB is due to the Mn-NO antibonding nature of the SOMO of the complex, and the VSSP contributions reflect this character. The almost cylindrical spin-density contributions are dominated by interactions between nitrogen and oxygen p_π_ orbitals and the corresponding metal d_π_ orbitals. These exaggerated VSSP contributions are effectively removed by the DE-corrections, as they are for MnO_3_ and [Mn(CN)_4_N]^−^. Adding only sc-corrections has a smaller effect, in particular for the damped models (Figure S8).

**Figure 5:**
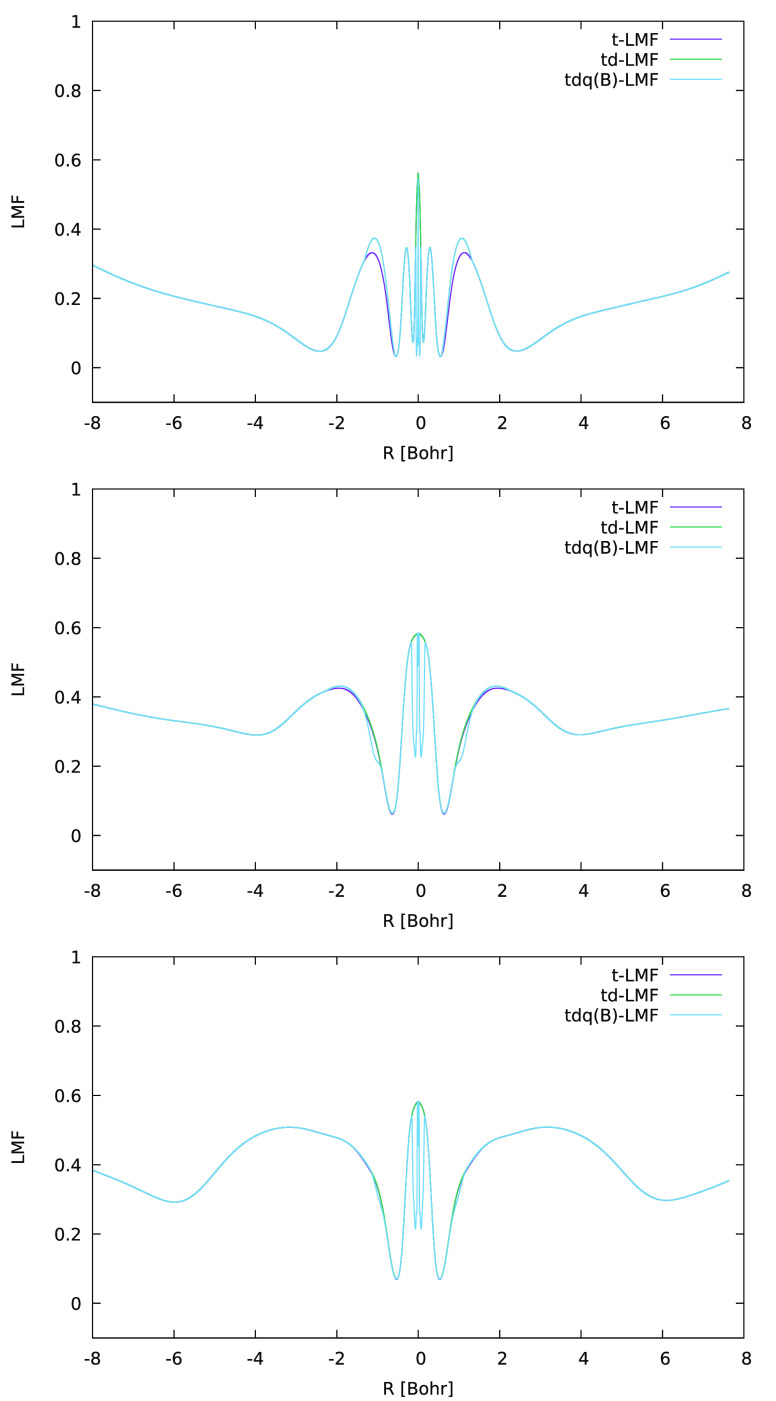
One-dimensional (1D) graphical comparison of t-LMF (ωLH22t), DE-corrected td-LMF (ωLH23td), and DE- and sc-corrected tdq-LMF (ωLH23tdB) for cluster-embedded [Mn(CN)_5_NO]^2–^ perpendicular to the Mn–NO bond at Mn (top), N (middle), and O (bottom).

The changes that the sc- and DE-corrections make to the LMF follow a similar pattern here as that discussed for MnO_3_ above (Figure 5): the DE-corrections enhance the EXX admixture locally in the metal valence shell, particularly perpendicular to the Mn–NO bond. In contrast, the sc-corrections locally diminish the EXX admixture on the NO nitrogen atom, also perpendicular to the bond, with very small effects on oxygen.

### 4.2 *A*_iso_ and Core–Shell Spin Polarization

*A*_iso_ of the complexes can be influenced indirectly by SSB due to the distorted valence-shell spin-density distribution caused by spin contamination, which potentially alters the CSSP of the 2s and 3s shells. But *A*_iso_ also depends crucially on internal mechanisms within the core and semicore–shells. We have previously observed that for the vast majority of functionals, from UHF all the way to LSDA calculations, the ratio between the 3s and 2s CSSP contributions to the ^55^Mn *A*_iso_ is typically in the range between −0.4 and −0.6 (the ratio differs somewhat for other metal centers).^24,25,30^ This holds for all oxidation and spin states of the complexes investigated. Only some highly parametrized Minnesota functionals gave 3s/2s ratios that diverged significantly from these expectations.^30^ We suspect that larger deviations likely signal unphysical effects of the given functional in the outer core region of the metal center. An analysis of this ratio might thus be an important tool when investigating new functionals. At the same time, most semilocal functionals underestimate the CSSP contributions overall, while increasing EXX admixture (and τ-dependent contributions in some highly parametrized meta-GGA functionals like M06-L^30^) tends to enhance CSSP.

#### 4.2.1 *A*_iso_ of MnO_3_

Due to the mixed Mn 4s/d_*z*^2^_ character of the SOMO in this doublet radical, *A*_iso_ is dominated by the direct SOMO contribution and thus large and positive, with negative CSSP contributions overall reducing its absolute value. Even though GHs with moderate EXX admixture or LHs/RSLHs without sc- or DE-corrections exhibit substantial SSB, their *A*_iso_ values tend to be reasonable (Table 2), and their 3s/2s ratios are unremarkable.

scLHs based on LH20t and scRSLHs without DE-corrections also give 3s/2s ratios close to LH20t or ωLH22t, respectively. But the sc-corrections do, to some extent, influence the overall CSSP contributions, due to the reduced SSB. This is most notable for scLH22ta, which exhibits an overall diminished CSSP contribution and, thus, a *A*_iso_ value that is too large. This is likely related to the appreciable reduction of SSB and of the resulting overall reduced valence spin density by this undamped scLH. Among the scLHs, only the simpler LSDA-based scLH21ct-SVWN-m exhibits an unusual 3s/2s ratio, signaling a distorted, unusually small 3s CSSP contribution (see Table S11 in the Supporting Information for more details). In this case, the overall CSSP contribution to *A*_iso_ becomes less negative.

Adding the DE-corrections for the scRSLHs reduces not only SSB and thereby affects CSSP indirectly, but it also distorts the 3s/2s ratio strikingly. In contrast to scLH21ct-SVWN-m, here, an overall larger negative CSSP contribution and, therefore, a (too) low *A*_iso_ is found (Table 2). The balance between the spin polarization of the 3s and 2s shells therefore seems to be distorted (the 3s contributions are determined notably by the radial orthogonality of the 3s and 2s orbitals both in the α and β spin channels^25^).

As this distortion of the 3s/2s ratio is a potentially undesirable aspect of the DE-corrections, we have examined the effect of parameter *h* that determines the magnitude of the DEC term (eq 8). Results are shown in Table 6, where *h* in the ωLH23td functional is varied from 0.0 (ωLH22t) to the nominal value used (12.0), in increments of 1.0. Most of the SSB and its effects on *A*_dip_ is already removed at *h* = 7.0. Then, the 3s/2s ratio is still in the usual range, and *A*_iso_ is still close to the value obtained without DE-correction. It appears that, as we increase *h* further, the enhanced EXX admixture near Mn starts to affect the balance between the 2s and 3s CSSP contributions, likely by too much. This is mainly due to a diminished 3s contribution, caused by the local enhancement of the EXX admixture around the metal center. We note in passing that the choice of *h* = 12.0 for the DE-corrected scRSLHs had been made based on the performance for the GMTKN55 main-group energetics test suite, but smaller values like *h* = 7.0 gave only marginally less-accurate results while improving on the BH76 barrier set, and *h* values of >12.0, in fact, gave numerically less stable calculations in that case.^20^

**Table 6:** Dependence of 3s/2s CSSP Ratio and *A*_iso_,^a^ as Well as SSB and *A*_dip_ (in MHz), for MnO_3_ on Parameter *h* in the DE-Correction in ωLH23td

	*A*_iso_			
*h*	3s/2s	total	*A*_dip_	⟨*S*^2^⟩
0.0	–0.63	1523.5	133.8	0.926
1.0	–0.61	1516.4	129.5	0.882
2.0	–0.60	1514.0	123.3	0.835
3.0	–0.57	1516.2	115.4	0.794
4.0	–0.54	1519.6	107.7	0.770
5.0	–0.51	1516.5	102.8	0.761
6.0	–0.47	1508.0	100.4	0.757
7.0	–0.43	1497.3	99.3	0.756
8.0	–0.39	1485.7	98.8	0.756
9.0	–0.35	1474.8	98.4	0.755
10.0	–0.31	1464.5	98.1	0.755
11.0	–0.26	1453.4	98.0	0.755
12.0	–0.23	1444.5	97.9	0.755
				
Exp^b^		1613	81	0.750

a For more details on the CSSP contributions, see Table S11 in the Supporting Information.

b Data taken from ref (80).

#### 4.2.2 *A*_iso_ of [Mn(CN)_4_N]^–^

For this complex, the negative CSSP contributions dominate *A*_iso_, which is, thus, overall negative (Table 4). CSSP is clearly underestimated by simple semilocal functionals like PBE. PBE0, the LH20t and LH23pt LHs, and the ωLH22t RSLH provide reasonable *A*_iso_ values, while the simpler LSDA-based LH12ct-SsifPW92 and LH12ct-SsirPW92 overshoot in this case. A reduction of SSB by the scLHs or by scRSLHs without DE-corrections leads to insufficiently negative *A*_iso_. This is most pronounced for the undamped scLH22ta. Adding the DE-corrections (ωLH23td and ωLH23tdX functionals) provides more negative *A*_iso_, overshooting the experimental value slightly. Similar to MnO_3_ above, however, then the 3s/2s CSSP ratio tends to again deviate dramatically from the usual range (this is true to a lesser extent for scLH21ct-SVWN-m). This is again due mainly to a very small 3s contribution (see Table S5 in the Supporting Information). We have therefore examined the effect of varying parameter *h* of the DE-correction also for this complex (Table S12 in the Supporting Information). A value of *h* = 7.0 again seems to be required to remove most of the SSB in this case. The 3s/2s ratio is then 0.36, i.e., slightly below the lower end of the usual range. Smaller values of *h* give larger ratios. We note, in passing, a very small discontinuity in the trend for *A*_iso_ around *h* = 4.0, which seems to reflect some numerical noise.

#### 4.2.3 *A*_iso_ of [Mn(CN)_5_NO]^2–^

When focusing on the embedded-cluster results for this complex, the observations are rather similar as with MnO_3_ or [Mn(CN)_4_N]^–^ (Table 5). While LH20t, LH23pt, or ωLH22t give insuffiently negative *A*_iso_, the LSDA-based LHs provide more negative values. The undamped scLHs lead to even less negative values, in particular scLH22ta, while correcting for SSB (see above). The damped scLHs and scRSLHs without DE-corrections remain relatively close to LH20t and ωLH22t, respectively. Adding DE-corrections leads to somewhat too negative *A*_iso_ and provides again unusual 3s/2s CSSP ratios (see Table 5).

Table 7 examines the effect of the magnitude of parameter *h* of the DE-correction on these results, for ωLH23td and ωLH23tdB. In the absence of sc-corrections (ωLH23td), an *h* value of 9.0 is required to remove most of the SSB. Then, the 3s/2s ratio (−0.24) is already much smaller in absolute value than the usual range. With sc-corrections (ωLH23tdB), *h* = 7.0 appears to suffice to remove most of the SSB, retaining a somewhat larger 3s/2s ratio (−0.30). There is obviously still some tradeoff between the reduction of SSB by DE-terms and the makeup of the CSSP contributions to *A*_iso_.

**Table 7:** Dependence of 3s/2s CSSP Ratio, *A*_iso_,^a^*A*_dip_, and ⟨*S*^2^⟩ for Cluster-Embedded [Mn(CN)_5_NO]^2–^ on Parameter *h* in the DE-Correction

		*A*_iso_			
	*h*	3s/2s	total	*A*_dip_	⟨*S*^2^⟩
ωLH23td	6.0	–0.41	–220.4	–100.5	0.873
	7.0	–0.37	–225.0	–105.7	0.839
	8.0	–0.31	–226.2	–113.6	0.798
	9.0	–0.24	–227.4	–120.6	0.773
	10.0	–0.19	–233.3	–123.2	0.766
	11.0	–0.15	–240.9	–124.2	0.765
	12.0	–0.11	–249.1	–124.5	0.764
					
ωLH23tdB	6.0	–0.35	–196.0	–114.4	0.791
	7.0	–0.30	–198.9	–119.9	0.773
	8.0	–0.25	–204.3	–122.8	0.766
	9.0	–0.21	–210.5	–124.6	0.762
	10.0	–0.16	–217.0	–125.7	0.761
	11.0	–0.12	–224.4	–126.2	0.760
	12.0	–0.08	–231.4	–126.5	0.760
					
Exp^b^			–219.5	–115.2	0.750

a For more details on the CSSP contributions, see Table S13 in the Supporting Information.

b Data taken from ref (71).

### 4.3 Results with Further Functionals

Two further scLHs, scLH23t-mBR and scLH23t-mBR-P, based on simplified constructions of *q*_*AC*_ analogous to ωLH23tE and ωLH23tP, respectively,^19^ have also been examined. Tables S2, S4, S6, S8, and S9 in the Supporting Information include results with these functionals. Due to their damped sc-factors, these scLHs only partially correct SSB in the VSSP cases, staying relatively close to the underlying LH20t and performing comparably to the also-damped scLH22t.

As the sc-factors had initially been formulated in analogy to the KP16/B13 model, we also examined the performance of this particular rung 4 functional for the four complexes (Table 8). It is clear, that KP16/B13 is very effective in removing SSB almost completely for the three VSSP cases. *A*_dip_ values appear to reflect this to some extent, albeit the effects appear to be overestimated for [Mn(CN)_4_N]^−^, and for (cluster-embedded) [Mn(CN)_5_NO]^2–^. Unfortunately, the *A*_iso_ values computed with KP16/B13 are very unrealistic and suggest overall large positive rather than negative CSSP contributions. This is true also for [Mn(CN)_4_]^2–^, i.e., independent of the presence or absence of VSSP. All core–shell contributions are completely unrealistic, including even the 1s contribution to *A*_iso_, and an extremely large positive 3s contribution. Therefore, the description of the core region with this functional is flawed.

**Table 8:** Examination of SSB and HFCs (in MHz; with Shell Breakdown of CSSP Contributions to *A*_iso_) for the Four Title Complexes with the KP16/B13 Functional

	*A*_iso_								
	1s	2s	3s	3s/2s	total	*A*_dip_	⟨*S*^2^⟩	*A*_iso_ (exp)	*A*_dip_ (exp)
[Mn(CN)_4_]^2–^	–132.8	–155.0	332.9	–2.15	77.6	–	8.762	–199^a^	–
MnO_3_	–44.6	19.5	401.7	20.56	2066.5	103.0	0.755	1613^b^	81^b^
[Mn(CN)_4_N]^−^	–148.2	–128.3	678.5	–5.29	339.6	–140.8	0.756	–276^c^	–122.4^c^
[Mn(CN)_5_NO]^2–^ (cluster)	–148.1	–163.1	725.7	–4.45	351.8	–131.0	0.755	–219.5^d^	–115.2^d^

a Data taken from ref (61).

b Data taken from ref (80).

c Data taken from ref (82).

d Data taken from ref (71).

Very little is known about the internal structure of the recent deep-neural-network functional DM21.^58^ However, in terms of the quantities that enter the neural network, it has been characterized as an RSLH. Given that it has been successfully trained to give small FSEs, it also seems justified to assume it to be a scRSLH. Therefore, its performance for the present Mn complexes is of interest. Results are provided in Table 9. Given the much-larger computational demand of the current implementation of DM21 in pySCF, compared to the scLH and scRSLH calculations in Turbomole, calculations on [Mn(CN)_5_NO]^2–^ were restricted to the isolated dianion, where SSB is even more pronounced than for the embedded-cluster model (see above). Based on the ⟨*S*^2^⟩ expectation values, DM21 is very effective in removing SSB, almost completely for MnO_3_ and [Mn(CN)_4_N]^−^. Even for the isolated [Mn(CN)_5_NO]^2–^, a value of 0.770 is achieved. Unfortunately, it has not been possible to extract *A*_dip_ values from these computations. Nonrelativistic computations of *A*_iso_ have been possible, based on the extraction of the spin density at the Mn nucleus. The correctness of these calculations has been verified by the essentially perfect reproduction of Turbomole *A*_iso_ results for simpler functionals (PBE, PBE0). However, the values computed with DM21 are completely unrealistic and extremely negative for all four complexes (see Table 9). This suggests that the core region is not described adequately by this functional. However, in contrast to the CSSP contributions with KP16/B13 that are too positive (see above), we suspect that the DM21 data reflect dramatically overestimated negative CSSP contributions. The poor performance of DM21 for *A*_iso_ may not be too surprising, since, except for total atomic energies, no quantities dominated by the atomic core regions have been employed in its training.^58^

**Table 9:** Computed ⟨*S*^2^⟩ and Spin Density (in a.u.) at the Mn Nucleus, and the Corresponding (Nonrelativistic) *A*_iso_ (in MHz), for the Four Title Complexes with the DM21 Functional

	ρ_Mn_^α–β^	*A*_iso_	⟨*S*^2^⟩	*A*_iso_(exp)
[Mn(CN)_4_]^2–^	–3.063	–680.2	8.758	–199^a^
MnO_3_	–0.933	–1035.5	0.753	1613^b^
[Mn(CN)_4_N]^−^	–2.751	–3054.0	0.754	–276^c^
[Mn(CN_5_)NO]^2–^ d	–2.767	–3071.9	0.770	–219.5^e^

a Data taken from ref (61).

b Data taken from ref (80).

c Data taken from ref (82).

d Results for isolated [Mn(CN_5_)NO]^2–^ based on BP86-optimized structure.

e Data taken from ref (71).

### 4.4 Relations to Other Studies

In the Introduction, we had referred to the differentiated views on the interrelation between SSB and electron correlation in transition-metal complexes in the recent work of Shee et al.,^31^ and it seems appropriate to come back to those arguments after the above analyses. One argument of that work has been that, except for weakly bound and antiferromagnetically coupled complexes, the observed SSB at single-reference levels is not usually a sign of substantial static correlation. To some extent, the present analyses agree with this statement, as we see that the SSB is not only diminished by sc-corrections but also by adding DE-corrections that, in fact, do not *decrease* but *increase* the EXX admixture locally. Of course, this also contradicts the usual observation that larger EXX admixtures automatically enhance SSB due to the overstabilization of high-spin contaminants at the UHF level.^83^ It is important to note that, for all three VSSP cases studied here (MnO_3_, [Mn(CN)_4_N]^−^, and [Mn(CN)_5_NO]^2–^), a GH with a low EXX admixture like B3LYP does not remove SSB completely. In the sense of ref (31), this would suggest that, for these three complexes, static correlation is, indeed, to some extent, an issue, and the SSB is not artificial. This, in turn, is then consistent with our finding that sc-corrections can, in fact, reduce SSB notably in the present context. The verdict is thus still open about a precise classification of such complexes regarding the importance of static correlation.

What we have also shown here, however, is that more traditional functionals cannot deliver an accurate description of the electronic structure of such systems. Semilocal functionals tend to give less SSB, except for some of the more highly parametrized meta-GGAs like M06-L or MN15-L.^30^ However, these functionals then suffer from more substantial DE, rendering the metal–ligand bonds too covalent, and delocalizing spin density too much onto the ligands. And they tend to underestimate the CSSP necessary for a proper description of the isotropic metal HFC.^24,25,30^ GHs and RSHs, and without specific precautions also LHs and RSLHs, can reduce DE and improve CSSP; however, for higher EXX admixtures, they can then enhance SSB (depending on SOMO character, see above). The best-performing functionals evaluated in this work, which are based on a more sophisticated mixing of exact and semilocal exchange-energy densities, seem to be the first ones that indeed escape this dilemma. This is due to the use of position-dependent EXX admixture, which, in scLHs, can even be locally negative in cases with strongly stretched bonds.^18,19^ It is important to re-emphasize that many bonds of 3d transition metals to ligand atoms or to other metal centers correspond to some kind of at least moderately stretched-bond situation,^4,5^ and sizable sc-corrections thus seem warranted.

The focus in the present work has been on the metal HFCs. Ligand HFCs will of course also be affected by SSB, but also by DE. For example, Remenyi and Kaupp have correlated the dependence of ligand HFCs in models for blue copper enzymes on EXX admixtures with GHs to g-tensors and metal HFCs.^84^ Larger EXX admixtures reduced metal–ligand covalency and thereby the delocalization of spin density onto a coordinated thiolate ligand (modeling cysteine). Similar results have been obtained for other copper systems.^7^ These are just a few relevant examples of the importance of the EXX admixture in reducing DE. Ligand HFCs are also crucial for the ligand NMR shifts in paramagnetic transition-metal complexes. Pritchard and Autschbach^85^ showed, for a series of paramagnetic acetylacetonate complexes, that the use of RSHs, with or without a system-dependent tuning of the range-separation parameter, can reduce DE and thereby improve the ligand ^1^H and ^13^C HFCs and contact shifts. However, such an approach will fail as soon as SSB becomes a serious issue. Then approaches like the scRSLHs evaluated here may become the method of choice. Work along these lines is in progress in our lab.

## 5 Conclusions

Spin-symmetry breaking (SSB) in quantum-chemical calculations on open-shell transition-metal complexes can have various reasons and can be detrimental for accurate results or a computational tool in other cases (e.g., for broken-symmetry treatments of antiferromagnetic couplings). Here, we have revisited earlier observations of SSB in a series of mononuclear manganese complexes in the context of the computation of their metal hyperfine couplings (HFCs). Substantially elevated ⟨*S*^2^⟩ expectation values with (global, local, range-separated, or range-separated local) hybrid functionals for species like MnO_3_, [Mn(CN)_4_N]^−^, or [Mn(CN)_5_NO]^2–^ go along with exaggerated valence-shell spin polarization (VSSP) and distorted dipolar metal HFCs. In addition, isotropic HFCs are strongly influenced by core–shell spin polarization (CSSP), the description of which usually requires some exact-exchange (EXX) admixture.

Therefore, a clear dilemma in the choice of DFT approaches in this area exists: simple semilocal functionals give low SSB but suffer from delocalization errors (DEs) and underestimated CSSP. Hybrids with larger EXX admixtures reduce DEs and improve CSSP but tend to suffer from exaggerated VSSP and thus from SSB. Here, we have applied novel local hybrid and range-separated local hybrid functionals with sc- and DE-correction factors. This allows a more granular use of EXX admixture in real space to address the noted dilemma. We found two strategies helpful to reduce or remove SSB while maintaining low DE: (a) recently proposed DE-corrections to the local mixing function (LMF) of a series of range-separated local hybrids for “abnormal open-shell regions” locally enhance EXX admixtures in the metal valence shell. To our initial surprise, this is a particularly effective means to reduce SSB in the sensitive complexes, by reducing unphysical delocalization of spin density onto the crucial ligand atoms. This reduces VSSP and thereby SSB. (b) Alternatively, sc-corrections applied to local hybrids or range-separated local hybrids locally reduce EXX admixtures at strongly bound ligand atoms. This reduces the VSSP directly in the valence space of these atoms, thereby diminishing SSB as well. The combined use of DE- and sc-corrections in a recent set of range-separated local hybrids (ωLH23tdX; X = E, B, P) is particularly effective in removing SSB. We find, however, that the DE-corrections in their standard parametrization tend to provide unusual ratios between the CSSP contributions from the metal 3s and 2s shells. Somewhat smaller prefactors *h* of the open-shell DE term in the LMF can retain the reduced SSB while providing a more standard description of CSSP.

Other recently proposed functionals incorporating sc-terms like the KP16/B13 functional or the DM21 deep-neural-network functional are also effective in reducing VSSP and SSB in the complexes studied here, but both of these functionals provide a completely unrealistic description of the core–shell contributions and, thus, very poor isotropic HFCs.

While the present work has focused on a few manganese complexes studied previously in the context of their metal HFCs, it also addresses the more general problem of SSB in transition-metal computations. It appears that modern functionals based on the EXX energy density provide tools to deal with challenging open-shell transition-metal complexes. Apart from EPR parameters and other spin-dependent properties, significant SSB can also be detrimental in the computational description of a variety of other structural and spectroscopic quantities. The modern density functionals evaluated here should also become promising tools in such contexts.

## Supporting Information Available

Additional data on hyperfine couplings and spin expectation values, as well as figures with spin-density distributions and local mixing functions (PDF)
