# Supplementary material for: Spin-Symmetry Breaking and Hyperfine Couplings in Transition-Metal Complexes Revisited Using Density Functionals Based on the Exact-Exchange Energy Density
Source: J Chem Theory Comput. 2024 Feb 27;20(5):2033–48. doi: 10.1021/acs.jctc.3c01422 (PMC10938646; doi:10.1021/acs.jctc.3c01422)
Supplement: Supplementary file 1 — ct3c01422_si_001.pdf [file ct3c01422_si_001.pdf]

Supporting Information: Spin-symmetry breaking and hyperfine couplings in transition-metal complexes revisited using density functionals based on the exact-exchange energy density

Artur Wodyński,* Bryan Lauw, Marc Reimann, and Martin Kaupp*

Technische Universität Berlin, Institut für Chemie, Theoretische Chemie/Quantenchemie, Sekr. C7, Straße des 17. Juni 135, D-10623, Berlin, Germany

E-mail: artur.wodynski@tu-berlin.de; martin.kaupp@tu-berlin.de

Table S1. Comparison of different functionals, including those having sc- and DE-corrections, for ^{55}Mn HFCs (in MHz; including a shell breakdown of A_{iso}) and SSB for $[Mn(CN)_4]^{2-}$

	DEC	$q_{AC}(\mathbf{r})$	A_{iso}					$\langle S^2 \rangle$
			1s	2s	3s	3s/2s	total	
PBE	−	−	-3.3	-326.3	138.5	-0.42	-96.1	8.764
PBE0	−	−	-6.0	-377.8	164.2	-0.43	-131.8	8.766
LH12ct-SsifPW92	−	−	-14.2	-353.6	131.6	-0.37	-169.7	8.761
LH12ct-SsirPW92	−	−	-13.9	-350.3	132.8	-0.38	-163.1	8.761
LH20t	−	−	-6.4	-323.5	144.3	-0.45	-129.6	8.760
ωLH22t	−	−	-5.7	-336.4	157.1	-0.47	-129.4	8.760
LH23pt	−	−	-10.9	-327.0	134.3	-0.41	-142.7	8.760
scLH21ct-SVWN-m	−	+	-11.0	-351.7	128.9	-0.37	-160.4	8.761
scLH22t	−	+	-5.6	-325.2	145.2	-0.45	-129.3	8.760
scLH22ta	−	+	2.5	-327.6	136.1	-0.42	-123.3	8.761
ωLH23tE	−	+	-5.7	-336.4	157.1	-0.47	-129.4	8.760
ωLH23tB	−	+	-4.1	-340.7	159.2	-0.47	-129.1	8.759
ωLH23tP	−	+	-5.7	-336.4	157.1	-0.47	-129.3	8.759
ωLH23td	+	−	1.9	-335.6	156.5	-0.47	-124.7	8.757
ωLH23tdE	+	+	1.9	-335.6	156.5	-0.47	-124.7	8.757
ωLH23tdB	+	+	8.5	-339.5	158.7	-0.47	-119.0	8.757
ωLH23tdP	+	+	5.9	-333.1	154.3	-0.46	-121.1	8.757
Exp. [S1]							-199	8.750

Table S2. Effects of different input structures on $\langle S^2 \rangle$ and A_{iso} (in MHz) for $Mn(CN)_4{}^{2-}$ with different functionals, and effects of COSMO solvent model in the HFC calculations

	B3LYP structure		BP86 structure		exp. structure [S1]			
					COSMO[a]			
	iso.	$\langle S^2 \rangle$	A_{iso}	$\langle S^2 \rangle$	A_{iso}	$\langle S^2 \rangle$	A_{iso}	$\langle S^2 \rangle$
PBE	-97.2	8.764	-93.9	8.766	-96.5	8.764	-96.1	8.764
PBE0	-133.9	8.766	-129.0	8.770	-132.3	8.766	-131.8	8.766
LH12ct-SsifPW92	-172.7	8.761	-166.4	8.764	-170.2	8.761	-169.7	8.761
LH12ct-SsirPW92	-166.0	8.761	-160.0	8.764	-163.7	8.761	-163.1	8.761
LH20t	-132.5	8.759	-126.7	8.762	-130.1	8.760	-129.6	8.760
ωLH22t	-132.2	8.759	-126.6	8.762	-129.8	8.759	-129.4	8.760
LH23pt	-145.9	8.760	-139.5	8.763	-143.2	8.760	-142.7	8.760
sLH21ct-SVWN-m	-162.8	8.761	-157.2	8.763	-160.9	8.761	-160.4	8.761
scLH22t	-132.2	8.759	-126.5	8.762	-129.8	8.759	-129.3	8.760
scLH22ta	-126.3	8.760	-120.4	8.763	-123.9	8.761	-123.3	8.761
scLH23t-mBR	-132.5	8.759	-126.7	8.762	-130.1	8.760	-129.6	8.760
scLH23t-mBR-P	-132.5	8.759	-126.7	8.762	-130.1	8.760	-129.6	8.760
ωLH23tE	-132.2	8.759	-126.6	8.762	-129.8	8.759	-129.4	8.760
ωLH23tB	-131.9	8.759	-126.3	8.762	-129.6	8.759	-129.1	8.759
ωLH23tP	-132.1	8.759	-126.5	8.762	-129.8	8.759	-129.3	8.759
ωLH23td	-127.0	8.757	-122.6	8.758	-125.0	8.757	-124.7	8.757
ωLH23tdE	-127.0	8.757	-122.6	8.758	-125.0	8.757	-124.7	8.757
ωLH23tdB	-121.1	8.757	-116.9	8.758	-119.2	8.757	-119.0	8.757
ωLH23tdP	-123.2	8.757	-119.2	8.758	-121.3	8.757	-121.1	8.757
Exp[S1]	-199	8.750	-199	8.750	-199	8.750	-199	8.750

[a]With $\varepsilon = 8.9$ for CH_2Cl_2 solvent.

Table S3. Comparison of different functionals, including those having sc- and DE-corrections, for ^{55}Mn HFCs (in MHz; including a shell breakdown of A_{iso}) and SSB of MnO_3.

	DEC	$q_{AC}(\mathbf{r})$	A_{iso}					A_{dip}	$\langle S^2 \rangle$
			1s	2s	3s	3s/2s	total		
PBE	−	−	15.2	-350.7	178.1	-0.51	1825.0	94.6	0.771
PBE0	−	−	12.4	-716.5	418.2	-0.58	1427.7	137.8	1.050
LH12ct-SsifPW92	−	−	19.6	-577.0	275.2	-0.48	1469.4	131.8	0.931
LH12ct-SsirPW92	−	−	19.2	-541.3	261.1	-0.48	1511.9	127.4	0.896
LH20t	−	−	34.1	-523.9	304.3	-0.58	1504.5	130.5	0.924
ωLH22t	−	−	29.7	-546.0	342.3	-0.63	1523.5	133.8	0.926
LH23pt	−	−	37.3	-537.1	304.8	-0.57	1454.7	131.6	0.954
scLH21ct-SVWN-m	−	+	41.9	-310.4	32.6	-0.11	1768.1	96.4	0.761
scLH22t	−	+	35.4	-353.5	205.4	-0.58	1612.9	102.1	0.772
scLH22ta	−	+	46.3	-312.3	148.6	-0.48	1733.4	95.0	0.763
ωLH23tE	−	+	31.8	-456.0	292.3	-0.64	1542.4	117.0	0.825
ωLH23tB	−	+	16.9	-364.2	234.7	-0.64	1635.9	103.0	0.770
ωLH23tP	−	+	31.6	-458.6	293.8	-0.64	1541.1	117.5	0.828
ωLH23td	+	−	-18.3	-270.6	61.8	-0.23	1444.5	97.9	0.755
ωLH23tdE	+	+	-18.0	-266.1	55.2	-0.21	1424.3	96.7	0.754
ωLH23tdB	+	+	-92.6	-263.5	43.2	-0.16	1410.1	93.4	0.755
ωLH23tdP	+	+	-17.3	-242.6	26.9	-0.11	1408.1	95.8	0.754
Exp.[S2]							1613	81	0.750

Table S4. Effects of different input structures on $\langle S^2 \rangle$ and Mn HFCs (in MHz) for MnO_3, with different functionals.

	B3LYP str.			BP86 str.			original str.[S3,4]		
	A_{iso}	A_{dip}	$\langle S^2 \rangle$	A_{iso}	A_{dip}	$\langle S^2 \rangle$	A_{iso}	A_{dip}	$\langle S^2 \rangle$
PBE	1858.7	93.8	0.770	1797.1	95.4	0.772	1825.0	94.6	0.771
PBE0	1469.7	134.8	1.014	1393.4	140.2	1.081	1427.7	137.8	1.050
LH12ct-SsifPW92	1513.2	129.2	0.908	1430.7	134.1	0.952	1469.4	131.8	0.931
LH12ct-SsirPW92	1550.4	126.0	0.886	1470.9	130.3	0.921	1511.9	127.4	0.896
LH20t	1546.6	127.6	0.900	1469.8	133.0	0.946	1504.5	130.5	0.924
ωLH22t	1565.9	130.6	0.902	1488.6	136.5	0.948	1523.5	133.8	0.926
LH23pt	1497.2	128.9	0.929	1419.7	134.0	0.978	1454.7	131.6	0.954
sLH21ct-sVWN-m	1803.0	95.6	0.761	1742.1	96.7	0.761	1768.1	96.4	0.761
scLH22t	1645.1	101.4	0.771	1583.3	102.4	0.772	1613.0	102.1	0.772
scLH22ta	1763.2	94.1	0.762	1704.9	95.3	0.762	1733.4	95.0	0.763
scLH23t-mBR	1560.6	113.3	0.819	1489.4	115.7	0.832	1521.8	114.6	0.825
scLH23t-mBR-P	1559.2	118.9	0.847	1485.5	122.6	0.872	1518.8	120.9	0.860
ωLH23tE	1582.1	115.6	0.818	1510.5	118.3	0.833	1542.4	117.0	0.825
ωLH23tB	1666.7	102.6	0.770	1606.3	103.2	0.770	1635.9	103.0	0.770
ωLH23tP	1580.8	116.2	0.821	1508.5	118.6	0.834	1541.1	117.5	0.828
ωLH23td	1481.2	97.1	0.754	1417.6	98.3	0.755	1444.6	97.9	0.755
ωLH23tdE	1459.6	96.1	0.754	1396.3	97.1	0.754	1424.3	96.7	0.754
ωLH23tdB	1435.1	93.1	0.754	1384.2	93.7	0.755	1410.1	93.4	0.755
ωLH23tdP	1445.8	95.2	0.754	1376.3	96.3	0.754	1408.1	95.8	0.754
Exp[S2]	1613.0	81	0.750	1613.0	81	0.750	1613	81	0.750

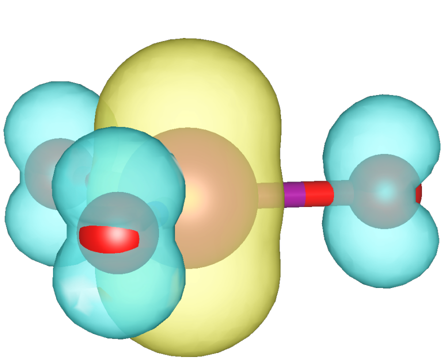

(a) ωLH22t

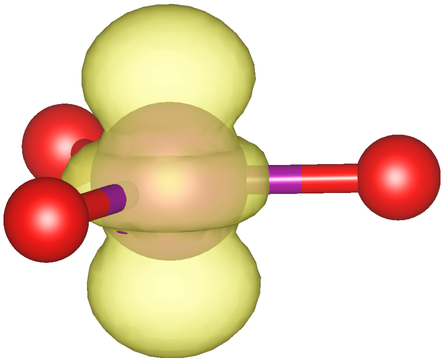

(b) ωLH23td

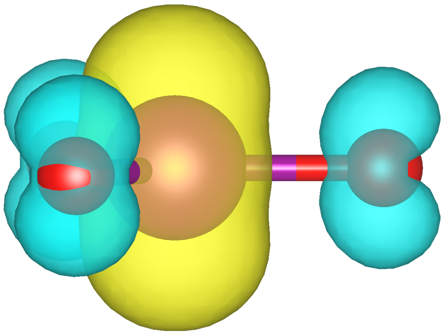

(c) LH20t

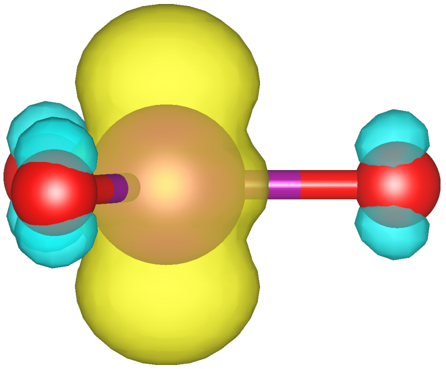

(d) scLH22t

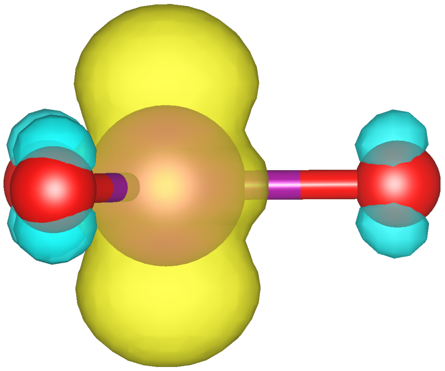

(e) ωLH23tB

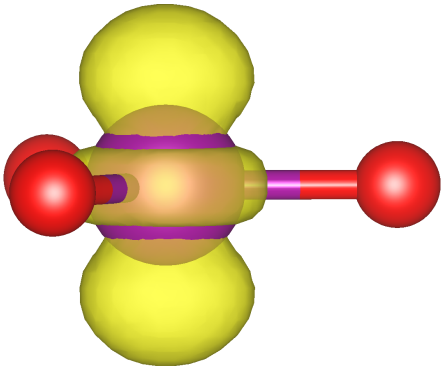

(f) ωLH23tdB

Figure S1. Spin density (isosurface ± 0.011 a.u.) for MnO_3 with selected functionals

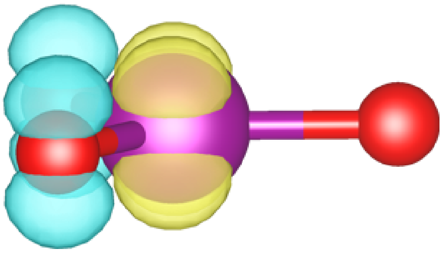

(a) MO19

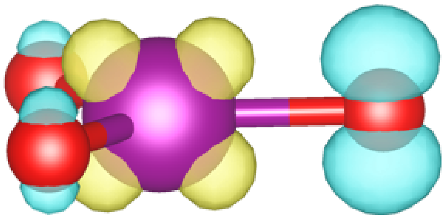

(b) MO20

Figure S2. Crucial MO spin densities (isosurface ± 0.011 a.u.) for MnO_3 with ωLH22t

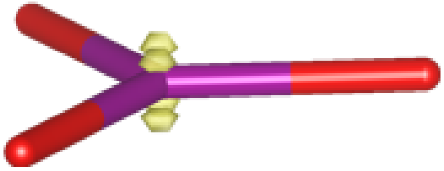

(a) MO19

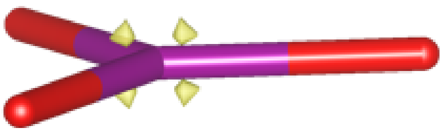

(b) MO20

Figure S3. Crucial MO spin densities (isosurface ± 0.011 a,u,) for MnO_3 with ωLH23td

Table S5. Comparison of different functionals, including those having sc- and DE-corrections, for ^{55}Mn HFCs (in MHz; including a shell breakdown of A_{iso}) and SSB of $[Mn(CN)_4N]^-$.

	DEC	$q_{AC}(\mathbf{r})$	A_{iso}					A_{dip}	$\langle S^2 \rangle$
			1s	2s	3s	3s/2s	total		
PBE	–	–	-2.5	-406.7	202.0	-0.50	-156.8	-115.1	0.774
PBE0	–	–	-8.7	-733.0	373.6	-0.51	-286.0	-111.4	0.981
LH12ct-SsifPW92	–	–	-23.2	-625.0	301.1	-0.48	-330.0	-116.8	0.903
LH12ct-SsirPW92	–	–	-22.0	-597.2	288.7	-0.48	-308.9	-117.8	0.880
LH20t	–	–	-9.2	-577.7	315.8	-0.55	-252.0	-115.6	0.903
ωLH22t	–	–	-8.3	-624.1	350.4	-0.56	-265.6	-117.0	0.933
LH23pt	–	–	-17.6	-607.2	310.8	-0.51	-289.3	-114.4	0.928
scLH21ct-SVWN-m	–	+	-13.0	-406.7	139.9	-0.34	-201.9	-124.1	0.768
scLH22t	–	+	-6.4	-460.0	244.7	-0.53	-182.6	-128.6	0.792
scLH22ta	–	+	3.7	-412.4	208.7	-0.51	-118.4	-124.0	0.774
ωLH23tE	–	+	-7.2	-532.5	297.8	-0.56	-219.1	-126.0	0.827
ωLH23tB	–	+	-4.3	-465.9	254.1	-0.55	-173.2	-134.3	0.783
ωLH23tP	–	+	-6.5	-469.1	261.1	-0.56	-179.8	-130.7	0.784
ωLH23td	+	–	6.5	-345.4	58.5	-0.17	-299.7	-139.3	0.756
ωLH23tdE	+	+	6.3	-340.8	55.2	-0.16	-295.1	-138.8	0.755
ωLH23tdB	+	+	20.6	-330.8	44.5	-0.13	-268.2	-139.4	0.754
ωLH23tdP	+	+	4.2	-307.8	18.8	-0.06	-295.8	-137.4	0.754
Exp. [S5]							-276	-122.4	0.750

Table S6. Effects of different input structures on $\langle S^2 \rangle$ and Mn HFCs (in MHz) for $[Mn(CN)_4N]^-$ and effects of COSMO solvent model in the HFC calculations.

	B3LYP str.			BP86 str.			original str.[S3,4]					
							COSMO[a]					
	A_{iso}	A_{dip}	$\langle S^2 \rangle$	A_{iso}	A_{dip}	$\langle S^2 \rangle$	A_{iso}	A_{dip}	$\langle S^2 \rangle$	A_{iso}	A_{dip}	$\langle S^2 \rangle$
PBE	-161.5	-116.6	0.773	-157.1	-114.2	0.774	-162.5	-115.3	0.776	-156.8	-115.1	0.774
PBE0	-283.7	-116.7	0.928	-286.5	-112.3	0.965	-301.9	-109.2	0.996	-286.0	-111.4	0.981
LH12ct-SsifPW92	-325.9	-120.8	0.872	-329.9	-117.0	0.896	-346.5	-114.8	0.918	-330.0	-116.8	0.903
LH12ct-SsirPW92	-308.8	-121.1	0.859	-311.1	-117.5	0.879	-326.1	-115.7	0.897	-308.9	-117.8	0.880
LH20t	-250.6	-120.0	0.868	-252.4	-116.1	0.893	-266.0	-113.8	0.915	-252.0	-115.6	0.903
ωLH22t	-263.0	-121.9	0.889	-267.0	-117.8	0.922	-281.6	-114.8	0.946	-265.6	-117.0	0.933
LH23pt	-285.6	-118.9	0.888	-289.0	-115.0	0.916	-304.9	-112.4	0.940	-289.3	-114.4	0.928
sLH21ct-SVWN-m	-206.0	-125.7	0.766	-199.5	-123.5	0.767	-206.6	-124.7	0.768	-201.9	-124.1	0.768
scLH22t	-187.3	-130.3	0.788	-180.9	-128.8	0.787	-187.1	-128.7	0.791	-182.6	-128.6	0.792
scLH22ta	-123.8	-125.5	0.772	-117.8	-123.6	0.772	-122.8	-124.3	0.774	-118.4	-124.0	0.774
scLH23t-mBR	-210.3	-125.8	0.806	-202.5	-123.9	0.805	-208.6	-123.5	0.809	-206.2	-123.7	0.812
scLH23t-mBR-P	-180.3	-128.3	0.783	-175.2	-126.6	0.782	-180.8	-127.1	0.783	-177.1	-126.8	0.785
ωLH23tE	-221.6	-128.4	0.816	-215.3	-126.4	0.819	-218.6	-125.6	0.820	-219.1	-126.0	0.827
ωLH23tB	-178.2	-135.6	0.781	-172.4	-134.4	0.780	-177.2	-134.4	0.781	-173.2	-134.3	0.783
ωLH23tP	-183.1	-132.1	0.782	-178.5	-130.5	0.782	-184.0	-130.6	0.783	-179.8	-130.7	0.784
ωLH22td	-301.3	-139.9	0.755	-298.2	-138.7	0.755	-301.0	-139.7	0.755	-299.7	-139.3	0.756
ωLH23tdE	-296.2	-139.4	0.755	-292.4	-138.2	0.755	-293.8	-139.2	0.754	-295.1	-138.8	0.755
ωLH23tdB	-270.3	-139.9	0.754	-267.2	-138.8	0.754	-268.9	-139.9	0.753	-268.2	-139.4	0.754
ωLH23tdP	-296.6	-138.1	0.754	-294.0	-136.9	0.754	-295.4	-138.2	0.754	-295.8	-137.4	0.754
Exp[S5]	-276	-122.4	0.750	-276	-122.4	0.750	-276	-122.4	0.750	-276	-122.4	0.750

[a]With $\varepsilon = 37.5$ for MeCN solvent.

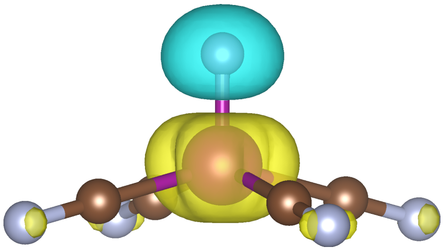

(a) ωLH22t

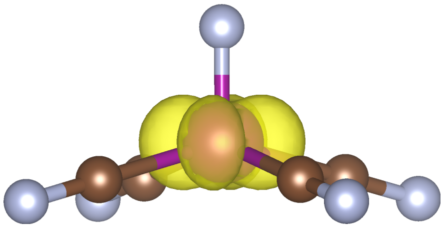

(b) ωLH23td

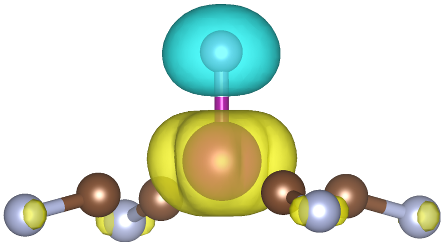

(c) LH20t

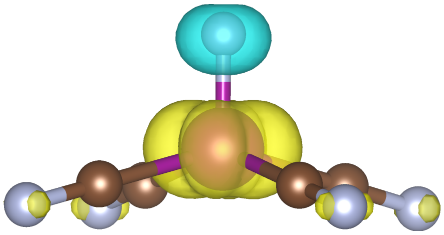

(d) scLH22t

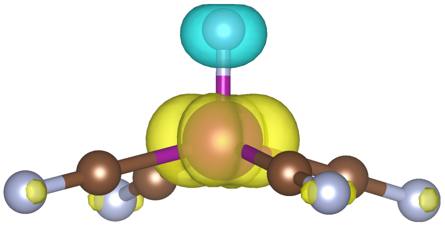

(e) ωLH23tB

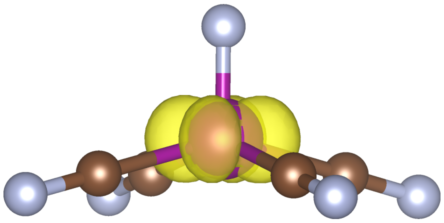

(f) ωLH23tdB

Figure S4. Spin density (isosurface ± 0.011 a.u.) for $[Mn(CN)_4N]^-$ with selected functionals

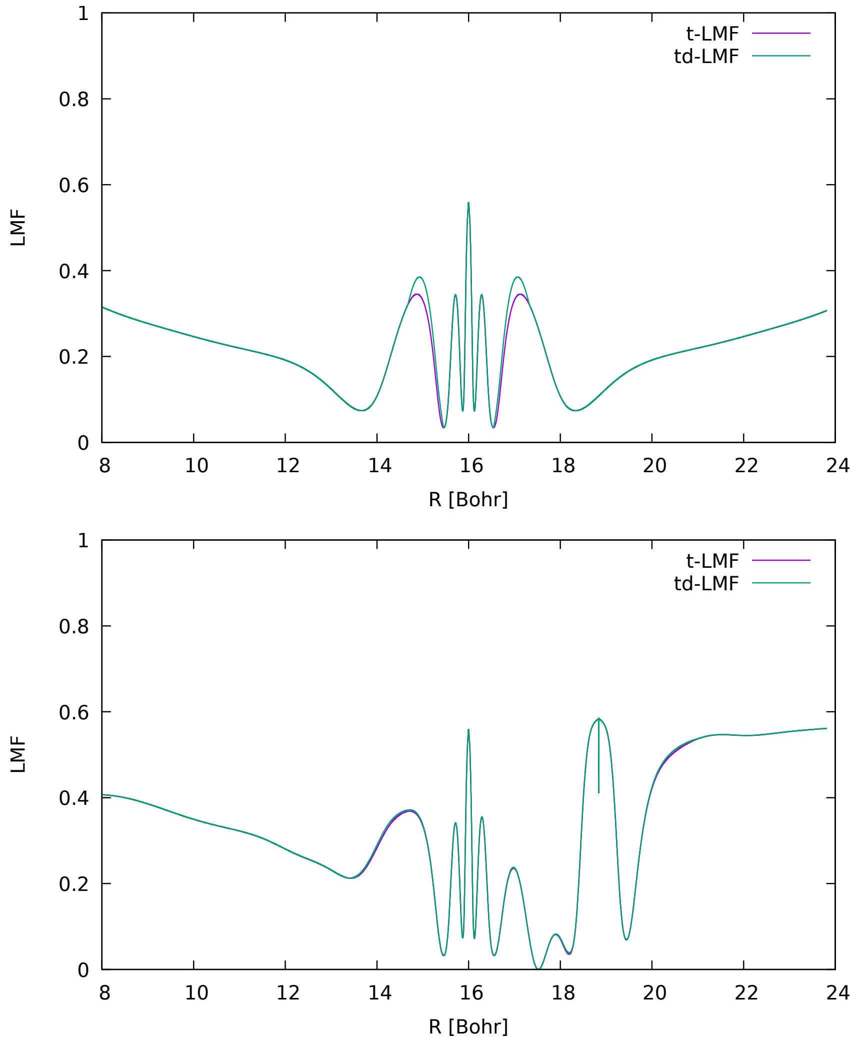

Figure S5. One-dimensional graphical comparison of t-LMF (ωLH22t) and DE-corrected td-LMF (ωLH23td) for $[Mn(CN)_4N]^-$ along lines passing through the Mn atom, perpendicular to the Mn-N(nitrido) bond (top) and along the Mn-N(nitrido) bond (bottom).

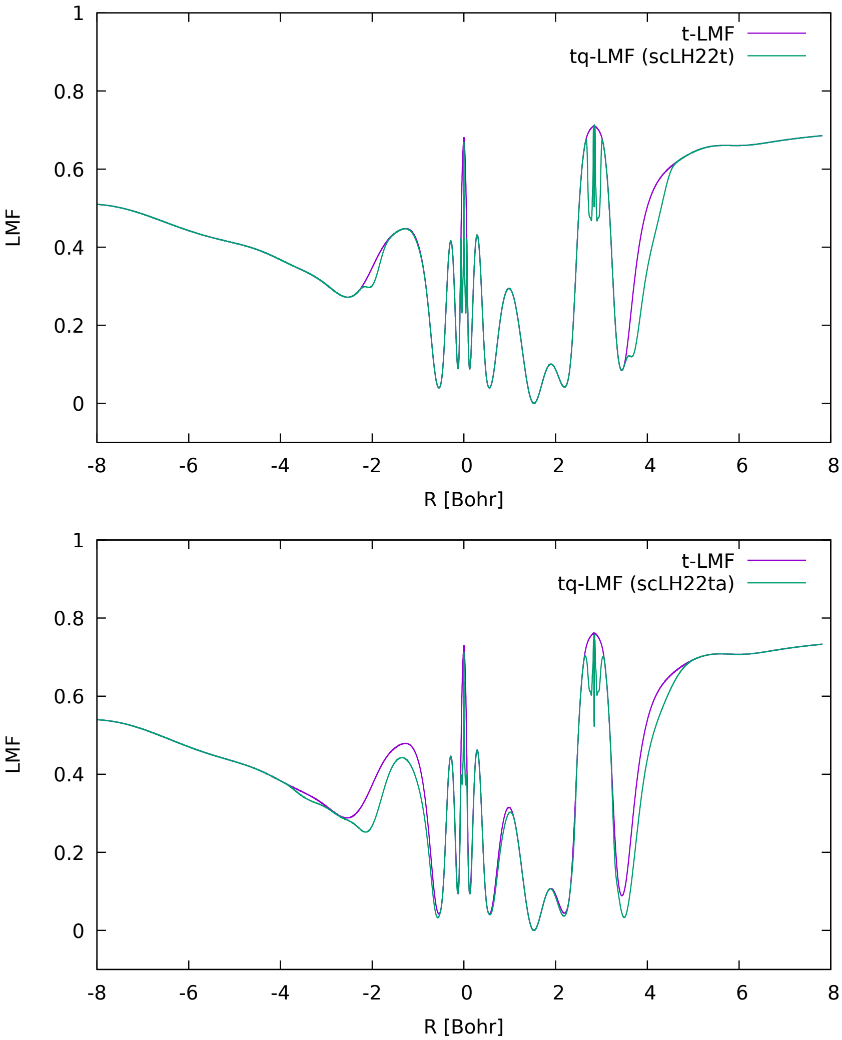

Figure S6. One-dimensional graphical comparison of t-LMF and sc-corrected tq-LMF for scLH22t (top) and scLH22ta (bottom) for $[Mn(CN)_4N]^-$ along the Mn-N(nitrido) bond.

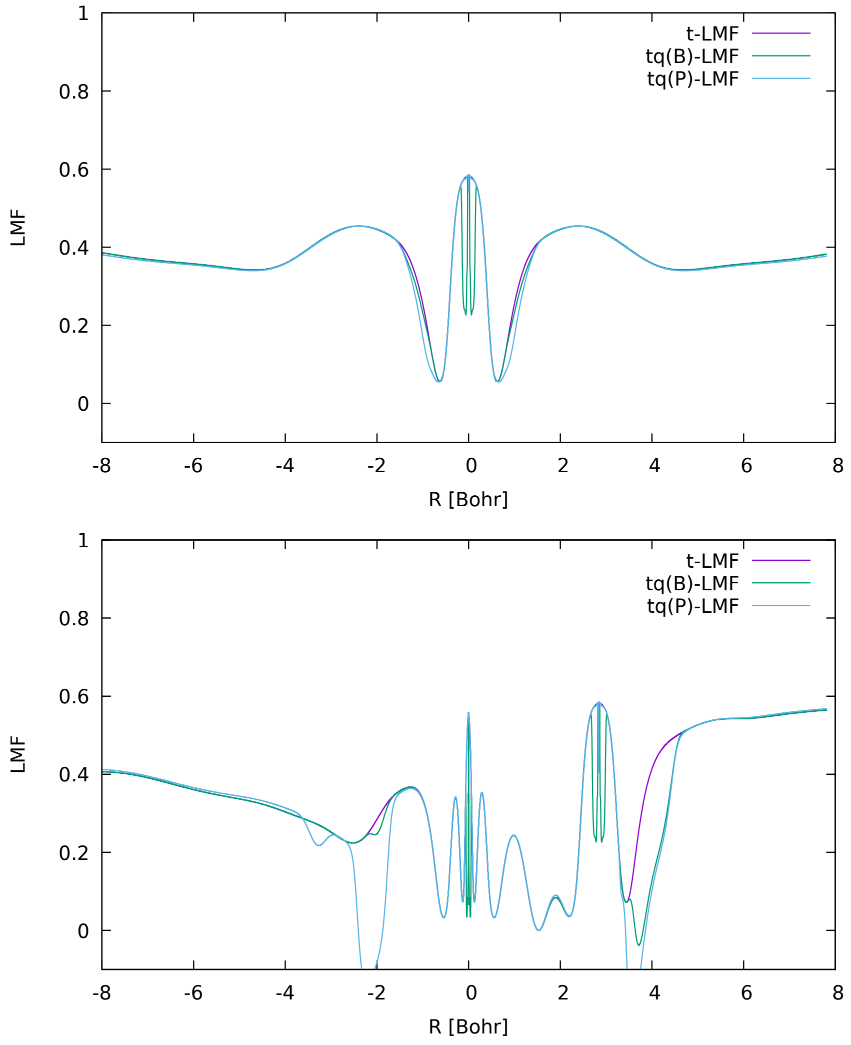

Figure S7. One-dimensional graphical comparison of t-LMF (ωLH22t) and sc-corrected tq-LMF (ωLH23tB and ωLH23tP) for $[Mn(CN)_4N]^-$ along lines passing through the nitrido nitrogen atom, perpendicular to the bond (top) and along the Mn-N(nitrido) bond (bottom).

Table S7. Comparison of different functionals, including those having sc- and DE-corrections for ^{55}Mn HFCs (in MHz; including a shell breakdown of A_{iso}) and SSB of $[Mn(CN)_5NO]^{2-}$ with cluster embedding[a] (see Computational Details).

	DEC	$q_{AC}(\mathbf{r})$	A_{iso}					A_{dip}	$\langle S^2 \rangle$
			1s	2s	3s	3s/2s	total		
PBE	–	–	-3.1	-390.0	180.9	-0.46	-104.6	-100.3	0.779
PBE0	–	–	-10.7	-702.3	352.2	-0.50	-191.2	-79.3	1.035
LH12ct-SsifPW92	–	–	-22.8	-593.5	272.9	-0.46	-233.8	-88.3	0.935
LH12ct-SsirPW92	–	–	-21.7	-570.8	263.0	-0.46	-219.6	-90.5	0.912
LH20t	–	–	-9.4	-562.2	300.4	-0.53	-160.4	-87.0	0.952
ωLH22t	–	–	-9.0	-612.1	335.4	-0.55	-170.3	-83.8	1.016
LH23pt	–	–	-17.4	-583.0	294.7	-0.51	-188.6	-84.7	0.972
sLH21ct-SVWN-m	+	–	-12.6	-377.2	127.7	-0.34	-153.9	-109.2	0.770
scLH22t	+	–	-7.8	-530.4	280.4	-0.53	-151.1	-92.8	0.898
scLH22ta	+	–	3.4	-377.6	192.2	-0.51	-68.9	-108.2	0.778
ωLH23tE	+	–	-9.0	-609.4	333.8	-0.55	-170.4	-84.3	1.011
ωLH23tB	+	–	-6.6	-589.3	320.8	-0.54	-161.7	-88.7	0.962
ωLH23tP	+	–	-8.9	-604.1	330.5	-0.55	-169.3	-85.2	1.000
ωLH22td	–	+	5.5	-345.1	38.8	-0.11	-249.1	-124.5	0.764
ωLH23tdE	+	+	5.5	-341.9	36.6	-0.11	-248.8	-125.1	0.763
ωLH23tdB	+	+	18.1	-331.5	27.9	-0.08	-231.5	-126.4	0.760
ωLH23tdP	+	+	3.8	-311.8	0.1	0.00	-256.3	-125.2	0.763
Exp. [S6]							-219.5	-115.2	0.750

Table S8. Effects of input structure (different gas-phase optimizations) and solvent model on HFCs (in MHz) and $\langle S^2 \rangle$ in $[Mn(CN)_5NO]^{2-}$

	B3LYP str.			BP86 str.			r2SCAN-3c str.			original str. [S3]		
ϵ (COSMO)	A_{iso}	A_{dip}	$\langle S^2 \rangle$	A_{iso}	A_{dip}	$\langle S^2 \rangle$	A_{iso}	A_{dip}	$\langle S^2 \rangle$	A_{iso}	A_{dip}	$\langle S^2 \rangle$
PBE												
–	-131.4	-99.6	0.838	-121.2	-101.9	0.809	-126.3	-100.7	0.823	-135.1	-96.6	0.858
4.0	-129.5	-101.8	0.825	-120.6	-103.1	0.803	-124.9	-102.5	0.814	-132.6	-99.3	0.840
78.3553	-128.4	-103.0	0.819	-120.1	-103.9	0.800	-124.3	-103.3	0.809	-130.8	-101.2	0.829
∞	-128.3	-103.1	0.818	-120.1	-103.9	0.800	-124.3	-103.4	0.809	-130.7	-101.2	0.829
PBE0												
–	-251.9	-52.5	1.520	-235.2	-57.8	1.400	-244.5	-54.6	1.467	-258.4	-49.1	1.590
4.0	-246.1	-57.6	1.433	-230.3	-62.5	1.325	-239.0	-59.6	1.383	-252.5	-54.3	1.498
78.3553	-241.5	-61.5	1.370	-226.3	-66.1	1.272	-234.5	-63.5	1.323	-247.6	-58.4	1.429
∞	-241.1	-61.8	1.366	-226.0	-66.3	1.268	-234.2	-63.8	1.319	-247.2	-58.7	1.424

Continuation of Table S8												
LH12ct-SsifPW92												
–	-327.2	-62.1	1.368	-300.8	-68.8	1.236	-315.0	-64.9	1.308	-338.2	-58.0	1.442
4.0	-316.5	-67.5	1.285	-291.7	-73.5	1.171	-304.9	-70.1	1.231	-327.0	-63.6	1.352
78.3553	-308.1	-71.5	1.227	-284.8	-77.0	1.125	-297.0	-73.9	1.178	-318.1	-67.9	1.287
∞	-307.5	-71.8	1.223	-284.3	-77.2	1.122	-296.4	-74.2	1.174	-317.5	-68.2	1.282
LH12ct-SsirPW92												
–	-308.6	-64.6	1.322	-282.5	-71.7	1.189	-296.6	-67.6	1.261	-319.4	-60.3	1.396
4.0	-298.1	-70.1	1.241	-273.9	-76.4	1.127	-286.8	-72.8	1.187	-308.5	-66.0	1.307
78.3553	-290.2	-74.1	1.185	-267.4	-79.8	1.085	-279.3	-76.7	1.136	-299.9	-70.4	1.244
∞	-289.6	-74.4	1.181	-266.9	-80.1	1.082	-278.7	-76.9	1.133	-299.3	-70.7	1.240
LH20t												
–	-224.0	-59.3	1.408	-205.5	-65.3	1.282	-215.7	-61.7	1.352	-231.1	-55.4	1.480
4.0	-218.2	-64.8	1.322	-200.7	-70.2	1.212	-210.2	-67.1	1.271	-225.0	-61.1	1.387
78.3553	-213.5	-69.0	1.260	-196.9	-73.9	1.163	-205.7	-71.2	1.214	-220.0	-65.6	1.319
∞	-213.1	-69.3	1.256	-196.6	-74.2	1.159	-205.4	-71.5	1.210	-219.6	-65.9	1.315

Continuation of Table S8												
ωLH22t												
–	-228.8	-55.7	1.502	-212.9	-60.8	1.386	-221.7	-57.7	1.450	-235.3	-52.1	1.571
4.0	-223.1	-61.3	1.411	-208.2	-65.8	1.310	-216.4	-63.1	1.364	-229.4	-57.9	1.474
78.3553	-218.5	-65.6	1.344	-204.4	-69.6	1.255	-211.9	-67.3	1.302	-224.4	-62.5	1.402
∞	-218.1	-65.9	1.339	-204.1	-69.9	1.251	-211.6	-67.6	1.297	-224.0	-62.8	1.397
scLH21ct-SVWN-m												
–	-189.3	-111.0	0.803	-176.5	-112.1	0.787	-182.7	-111.5	0.795	-192.7	-109.7	0.810
4.0	-186.0	-112.6	0.796	-175.1	-113.0	0.784	-180.5	-112.6	0.790	-189.1	-111.3	0.802
78.3553	-183.8	-113.6	0.791	-174.0	-113.5	0.782	-178.8	-113.5	0.786	-186.4	-112.5	0.796
∞	-183.7	-113.6	0.791	-173.9	-113.6	0.781	-178.6	-113.5	0.786	-186.2	-112.6	0.796
scLH22t												
–	-222.4	-60.9	1.381	-203.0	-67.8	1.245	-213.7	-63.7	1.319	-229.8	-56.8	1.455
4.0	-215.3	-67.2	1.284	-196.7	-73.6	1.165	-206.9	-69.8	1.230	-222.6	-63.2	1.352
78.3553	-209.3	-72.1	1.213	-191.4	-78.2	1.107	-201.0	-74.7	1.163	-216.3	-68.4	1.274
∞	-208.8	-72.5	1.208	-191.0	-78.5	1.102	-200.6	-75.1	1.158	-215.8	-68.8	1.268

Continuation of Table S8												
scLH22ta												
–	-101.4	-104.2	0.844	-86.6	-108.5	0.807	-93.6	-106.5	0.824	-108.6	-100.3	0.872
4.0	-97.5	-107.3	0.824	-86.0	-109.8	0.800	-91.5	-108.6	0.811	-102.2	-104.7	0.841
78.3553	-96.0	-108.8	0.816	-85.7	-110.6	0.796	-90.8	-109.6	0.806	-98.9	-107.2	0.826
∞	-95.9	-108.9	0.815	-85.7	-110.7	0.796	-90.8	-109.7	0.805	-98.7	-107.4	0.825
scLH23t-mBR												
–	-224.0	-59.3	1.408	-205.4	-65.8	1.276	-215.7	-61.9	1.349	-231.1	-55.4	1.480
4.0	-218.2	-65.4	1.315	-200.4	-71.7	1.195	-210.0	-68.2	1.257	-224.9	-62.3	1.371
78.3553	-213.3	-70.1	1.247	-196.6	-75.3	1.147	-205.5	-72.4	1.199	-219.8	-66.8	1.303
∞	-213.0	-70.4	1.243	-196.3	-75.5	1.144	-205.2	-72.7	1.195	-219.4	-67.1	1.298
scLH23t-mBR-P												
–	-223.9	-60.0	1.396	-205.1	-66.3	1.268	-215.5	-62.6	1.339	-231.0	-56.1	1.469
4.0	-217.8	-65.7	1.308	-200.1	-71.4	1.197	-209.7	-68.1	1.257	-224.7	-62.0	1.374
78.3553	-212.9	-70.0	1.246	-196.1	-75.2	1.147	-205.1	-72.3	1.199	-219.4	-66.6	1.304
∞	-212.5	-70.4	1.241	-195.8	-75.5	1.143	-204.7	-72.6	1.194	-219.1	-66.9	1.299

Continuation of Table S8												
ωLH23tE												
–	-228.9	-55.9	1.497	-213.0	-61.3	1.380	-221.8	-58.0	1.446	-235.4	-52.4	1.567
4.0	-223.3	-62.3	1.397	-208.3	-67.0	1.295	-216.4	-64.2	1.350	-229.5	-58.9	1.461
78.3553	-218.6	-66.7	1.331	-204.4	-70.8	1.241	-212.0	-68.4	1.288	-224.5	-63.6	1.387
∞	-218.2	-67.0	1.326	-204.2	-71.1	1.237	-211.7	-68.7	1.284	-224.1	-64.0	1.382
ωLH23tB												
–	-228.3	-56.7	1.481	-211.6	-62.4	1.359	-220.8	-59.0	1.428	-235.0	-53.0	1.553
4.0	-221.7	-62.8	1.384	-205.9	-67.9	1.276	-214.5	-64.9	1.335	-228.3	-59.3	1.450
78.3553	-216.1	-67.6	1.311	-201.1	-72.3	1.215	-209.1	-69.6	1.266	-222.5	-64.3	1.371
∞	-215.7	-68.0	1.306	-200.7	-72.6	1.211	-208.7	-69.9	1.261	-222.1	-64.7	1.365
ωLH23tP												
–	-229.3	-56.6	1.486	-213.2	-62.1	1.366	-222.1	-58.8	1.433	-235.9	-53.0	1.556
4.0	-223.3	-62.5	1.391	-208.1	-67.4	1.287	-216.4	-64.5	1.344	-229.7	-59.1	1.455
78.3553	-218.3	-67.1	1.322	-204.0	-71.4	1.231	-211.7	-68.9	1.279	-224.4	-64.0	1.380
∞	-217.9	-67.4	1.317	-203.7	-71.7	1.227	-211.3	-69.2	1.275	-224.0	-64.3	1.374

Continuation of Table S8

ωLH23td												
–	-329.8	-60.3	1.432	-318.0	-69.2	1.275	-324.4	-63.7	1.366	-335.1	-55.5	1.515
4.0	-323.8	-69.1	1.303	-311.4	-79.1	1.146	-318.3	-72.9	1.237	-329.4	-64.1	1.384
78.3553	-317.6	-76.9	1.197	-303.0	-89.5	1.028	-311.7	-81.3	1.129	-323.7	-71.5	1.278
∞	-317.0	-77.5	1.189	0.0	1.0	-311.058	-82.1	1.1	-323.158	-72.1	1.3	0.000
ωLH23tdE												
–	-329.8	-60.3	1.432	-318.2	-69.9	1.266	-324.5	-63.8	1.364	-335.1	-55.5	1.515
4.0	-324.2	-70.5	1.285	-276.5	-122.0	0.793	-318.6	-74.7	1.215	-330.0	-65.3	1.368
78.3553	-317.7	-79.0	1.172	-269.3	-126.3	0.777	-311.2	-84.4	1.095	-324.1	-73.5	1.254
∞	-317.1	-79.7	1.164	-268.9	-126.5	0.776	-310.5	-85.2	1.086	-323.6	-74.1	1.246
ωLH23tdB												
–	-315.6	-61.3	1.413	-248.4	-130.7	0.766	-310.5	-65.1	1.343	-321.2	-56.0	1.505
4.0	-308.7	-71.9	1.260	-246.6	-130.9	0.764	-250.6	-129.5	0.769	-314.8	-65.8	1.355
78.3553	-252.6	-128.6	0.773	-245.2	-130.9	0.763	-248.0	-130.2	0.767	-307.5	-75.2	1.225
∞	-252.2	-128.8	0.773	-245.1	-130.9	0.763	-247.9	-130.2	0.766	-266.1	-118.9	0.813

Continuation of Table S8

ωLH23tdP

–	-340.2	-60.4	1.431	-285.2	-122.5	0.792	-335.4	-63.9	1.364	-346.0	-55.4	1.519
4.0	-334.5	-70.0	1.291	-276.7	-127.6	0.773	-328.0	-74.8	1.213	-340.5	-64.7	1.377
78.3553	-326.6	-79.5	1.164	-274.1	-128.2	0.770	-285.2	-121.0	0.798	-333.9	-73.3	1.255
∞	-325.9	-80.4	1.154	-274.0	-128.3	0.770	-284.9	-121.2	0.797	-333.3	-74.0	1.246

Table S9. Effects of input structure (different optimizations with COSMO, $\varepsilon = 4.0$) and solvent model on HFCs (in MHz) and $\langle S^2 \rangle$ in $[Mn(CN)_5NO]^{2-}$

	B3LYP str.			BP86 str.			r2SCAN-3c str.		
ϵ (COSMO)	A_{iso}	A_{dip}	$\langle S^2 \rangle$	A_{iso}	A_{dip}	$\langle S^2 \rangle$	A_{iso}	A_{dip}	$\langle S^2 \rangle$
PBE									
–	-127.7	-100.6	0.826	-119.5	-101.3	0.807	-123.5	-100.8	0.816
4.0	-126.0	-102.7	0.815	-118.8	-102.6	0.801	-122.2	-102.5	0.808
78.3553	-125.2	-103.6	0.810	-118.3	-103.4	0.798	-121.6	-103.4	0.804
∞	-125.2	-103.7	0.810	-118.3	-103.4	0.798	-121.6	-103.5	0.803
PBE0									
–	-246.3	-54.6	1.470	-233.4	-57.8	1.389	-240.3	-55.9	1.433
4.0	-239.9	-60.2	1.378	-227.9	-62.9	1.309	-234.2	-61.3	1.345
78.3553	-234.7	-64.4	1.312	-223.4	-66.8	1.252	-229.2	-65.5	1.282
∞	-234.3	-64.8	1.308	-223.1	-67.1	1.248	-228.8	-65.8	1.277
LH12ct-SsifPW92									
–	-317.6	-64.7	1.314	-297.3	-68.9	1.224	-307.8	-66.5	1.272
4.0	-306.0	-70.5	1.229	-287.4	-74.0	1.155	-296.8	-72.0	1.193
78.3553	-297.1	-74.8	1.170	-279.8	-77.8	1.106	-288.3	-76.1	1.138
∞	-296.4	-75.1	1.166	-279.3	-78.0	1.103	-287.6	-76.4	1.134
LH12ct-SsirPW92									
–	-299.2	-67.3	1.268	-279.2	-71.8	1.178	-289.5	-69.2	1.226
4.0	-288.0	-73.1	1.186	-269.7	-76.9	1.112	-278.9	-74.7	1.150
78.3553	-279.5	-77.4	1.130	-262.6	-80.6	1.067	-270.9	-78.8	1.098
∞	-278.8	-77.7	1.126	-262.1	-80.8	1.064	-270.3	-79.1	1.095

Continuation of Table S9									
LH20t									
–	-217.8	-61.7	1.356	-203.3	-65.4	1.272	-210.9	-63.2	1.317
4.0	-211.4	-67.6	1.267	-197.9	-70.7	1.197	-204.9	-68.9	1.233
78.3553	-206.2	-72.2	1.204	-193.6	-74.7	1.144	-200.0	-73.3	1.174
∞	-205.9	-72.5	1.199	-193.3	-75.0	1.140	-199.6	-73.6	1.170
ωLH22t									
–	-223.4	-57.8	1.451	-211.2	-60.9	1.376	-217.7	-59.0	1.417
4.0	-217.1	-63.9	1.356	-205.9	-66.3	1.294	-211.7	-64.9	1.327
78.3553	-211.9	-68.6	1.286	-201.5	-70.5	1.234	-206.8	-69.4	1.260
∞	-211.5	-69.0	1.281	-201.1	-70.8	1.230	-206.4	-69.7	1.256
LH23pt									
–	-254.5	-60.3	1.379	-238.7	-63.8	1.296	-247.0	-61.7	1.340
4.0	-246.1	-66.2	1.287	-231.6	-69.1	1.218	-239.0	-67.4	1.254
78.3553	-239.5	-70.7	1.222	-225.9	-73.1	1.163	-232.7	-71.8	1.192
∞	-239.0	-71.1	1.217	-225.5	-73.4	1.159	-232.2	-72.1	1.188
sLH21ct-SVWN-m									
–	-184.5	-111.3	0.798	-173.9	-111.4	0.787	-178.8	-111.2	0.792
4.0	-181.7	-112.6	0.792	-172.3	-112.3	0.783	-176.7	-112.3	0.787
78.3553	-179.6	-113.6	0.787	-171.2	-112.8	0.780	-174.9	-113.2	0.783
∞	-179.5	-113.7	0.787	-171.2	-112.9	0.780	-174.8	-113.2	0.783
scLH22t									
–	-215.8	-63.5	1.327	-200.6	-67.9	1.235	-208.6	-65.2	1.285
4.0	-208.0	-70.3	1.228	-193.6	-74.1	1.149	-201.0	-71.9	1.191
78.3553	-201.4	-75.6	1.155	-187.7	-79.1	1.087	-194.6	-77.1	1.121
∞	-200.9	-76.0	1.150	-187.2	-79.4	1.083	-194.1	-77.5	1.116

Continuation of Table S9									
scLH22ta									
–	-96.0	-105.7	0.830	-84.7	-107.8	0.806	-89.9	-106.7	0.817
4.0	-93.1	-108.3	0.814	-83.8	-109.2	0.799	-88.2	-108.7	0.806
78.3553	-92.1	-109.5	0.808	-83.5	-110.1	0.795	-87.6	-109.7	0.801
∞	-92.1	-109.6	0.807	-83.5	-110.1	0.794	-87.6	-109.8	0.800
scLH23t-mBR									
–	-217.8	-61.7	1.356	-203.2	-65.8	1.266	-210.9	-63.2	1.318
4.0	-211.3	-68.1	1.261	-197.5	-72.0	1.181	-204.7	-69.6	1.225
78.3553	-206.1	-73.0	1.193	-193.2	-76.0	1.129	-199.6	-74.4	1.161
∞	-205.7	-73.4	1.189	-192.9	-76.3	1.126	-199.3	-74.7	1.157
scLH23t-mBR-P									
–	-217.6	-62.4	1.345	-202.9	-66.4	1.258	-210.6	-64.0	1.305
4.0	-210.9	-68.5	1.254	-197.2	-71.9	1.181	-204.3	-69.9	1.219
78.3553	-205.6	-73.2	1.190	-192.7	-75.9	1.128	-199.2	-74.4	1.159
∞	-205.2	-73.5	1.185	-192.4	-76.2	1.124	-198.8	-74.7	1.155
ωLH23tE									
–	-223.4	-57.9	1.451	-211.1	-61.3	1.370	-217.7	-59.2	1.415
4.0	-217.2	-64.6	1.347	-205.9	-67.4	1.279	-211.8	-65.6	1.317
78.3553	-212.0	-69.5	1.276	-201.5	-71.6	1.221	-206.8	-70.4	1.249
∞	-211.6	-69.8	1.271	-201.1	-71.9	1.216	-206.3	-70.8	1.243
ωLH23tB									
–	-222.4	-59.0	1.430	-209.6	-62.5	1.349	-216.5	-60.4	1.394
4.0	-215.1	-65.6	1.328	-203.2	-68.5	1.259	-209.4	-66.8	1.296
78.3553	-208.9	-70.9	1.251	-197.7	-73.3	1.193	-203.3	-71.9	1.223
∞	-208.4	-71.3	1.246	-197.3	-73.6	1.189	-202.9	-72.3	1.217

Continuation of Table S9									
ωLH23tP									
–	-223.7	-58.8	1.436	-211.3	-62.1	1.357	-217.9	-60.1	1.400
4.0	-217.1	-65.1	1.337	-205.6	-67.8	1.272	-211.5	-66.2	1.307
78.3553	-211.6	-70.0	1.266	-200.9	-72.2	1.211	-206.3	-70.9	1.239
∞	-211.1	-70.4	1.261	-200.6	-72.5	1.207	-205.9	-71.3	1.234
ωLH23td									
–	-325.1	-63.6	1.370	-316.0	-69.6	1.264	-321.0	-65.8	1.327
4.0	-318.1	-73.6	1.229	-308.3	-80.7	1.121	-313.3	-76.5	1.181
78.3553	-310.0	-83.1	1.110	0.0	0.9	-304.603	-86.9	1.1	0.000
∞	-309.3	-83.9	1.101	-271.9	-120.3	0.795	-303.8	-87.8	1.047
ωLH23tdE									
–	-325.1	-63.6	1.370	-316.0	-69.9	1.260	-321.0	-65.8	1.327
4.0	-318.1	-74.9	1.214	-273.8	-121.7	0.791	-313.6	-78.2	1.161
78.3553	-309.6	-85.4	1.085	-266.2	-126.2	0.774	-275.0	-120.3	0.799
∞	-308.9	-86.3	1.075	-265.8	-126.4	0.774	-274.6	-120.4	0.798
ωLH23tdB									
–	-311.0	-65.0	1.348	-246.7	-129.9	0.766	-306.4	-67.8	1.297
4.0	-262.0	-120.3	0.802	-244.7	-130.1	0.764	-247.9	-129.2	0.768
78.3553	-248.9	-129.1	0.770	-243.2	-130.2	0.763	-245.5	-129.8	0.766
∞	-248.6	-129.2	0.769	-243.1	-130.2	0.763	-245.3	-129.8	0.765
ωLH23tdP									
–	-336.1	-63.6	1.371	-282.7	-122.1	0.791	-331.4	-66.3	1.321
4.0	-327.6	-75.3	1.209	-274.3	-127.0	0.773	-284.3	-120.4	0.799
78.3553	-285.7	-119.5	0.805	-271.6	-127.7	0.770	-275.5	-126.1	0.776
∞	-285.3	-119.8	0.804	-271.4	-127.7	0.769	-275.1	-126.2	0.776

Table S10. Comparison of different functionals with sc- and DE-corrections for $\langle S^2 \rangle$ and HFCs (in MHz) of cluster-embedded $[Mn(CN)_5NO]^{2-}$ using a r^2SCAN-3c optimized structure.

	$q_{AC}(\mathbf{r})$	DEC	A_{iso}	A_{dip}	$\langle S^2 \rangle$
LH20t	−	−	-164.0	-87.0	0.963
ωLH22t	−	−	-173.5	-83.9	1.025
ωLH23tE	+	−	-173.7	-84.4	1.021
ωLH23tB	+	−	-164.7	-89.0	0.969
ωLH23tP	+	−	-172.6	-85.3	1.010
sLH21ct-SVWN-m	+	−	-157.9	-110.5	0.771
scLH22t	+	−	-155.1	-92.5	0.910
scLH22ta	+	−	-71.3	-109.5	0.779
ωLH23td	−	+	-251.0	-124.5	0.767
ωLH23tdE	+	+	-250.4	-125.3	0.765
ωLH23tdB	+	+	-232.5	-127.0	0.760
ωLH23tdP	+	+	-258.1	-125.5	0.764
Exp.[S6]			-219.5	-115.2	0.750

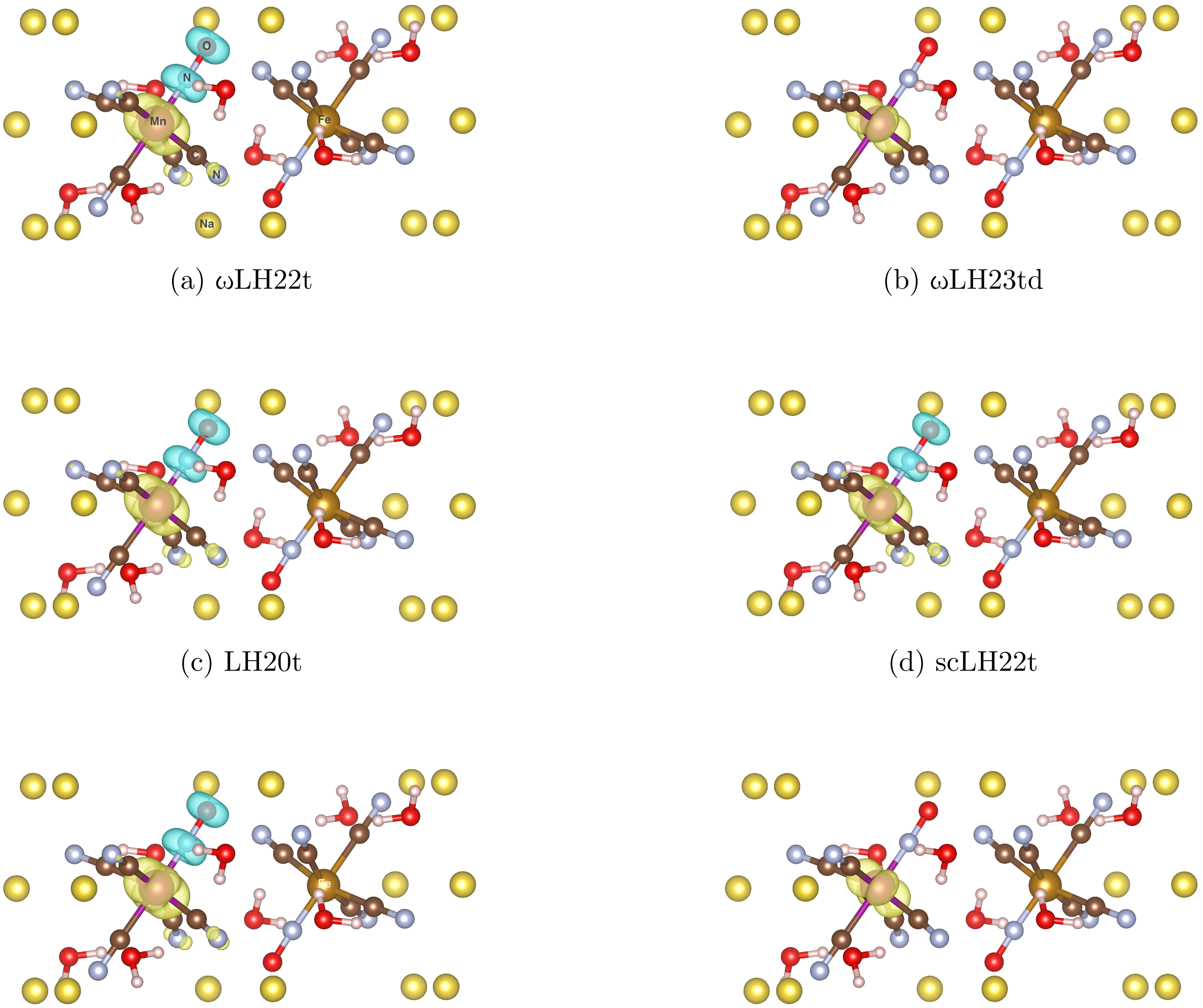

(a) ωLH22t (b) ωLH23td

(c) LH20t (d) scLH22t

(e) ωLH23tB (f) ωLH23tdB

Figure S8. Spin density (isosurface ± 0.011 a.u.) for cluster-embedded $[Mn(CN)_5NO]^{2-}$ with selected functionals

Table S11. Dependence of CSSP and A_{iso}, as well as SSB and A_{dip} (in MHz) for MnO_3, on parameter h in the DE-correction in ωLH23td.

h	A_{iso}					A_{dip}	$\langle S^2 \rangle$
	1s	2s	3s	3s/2s	total		
0.0	29.7	-546.0	342.3	-0.63	1523.5	133.8	0.926
1.0	25.4	-505.3	309.6	-0.61	1516.4	129.5	0.882
2.0	21.1	-455.2	271.2	-0.60	1514.0	123.3	0.835
3.0	17.0	-399.4	228.7	-0.57	1516.2	115.4	0.794
4.0	12.7	-351.8	191.0	-0.54	1519.6	107.7	0.770
5.0	8.2	-323.4	164.2	-0.51	1516.5	102.8	0.761
6.0	3.7	-308.2	145.0	-0.47	1508.0	100.4	0.757
7.0	-0.6	-298.8	129.0	-0.43	1497.3	99.3	0.756
8.0	-4.7	-292.3	114.8	-0.39	1485.7	98.8	0.756
9.0	-8.5	-285.8	100.8	-0.35	1474.8	98.4	0.755
10.0	-12.1	-279.5	86.9	-0.31	1464.5	98.1	0.755
11.0	-15.5	-274.2	72.5	-0.26	1453.4	98.0	0.755
12.0	-18.3	-270.6	61.8	-0.23	1444.5	97.9	0.755
Exp.[S2]					1613	81	0.750

Table S12. Dependence of CSSP and A_{iso}, as well as SSB and A_{dip} (in MHz) for $[Mn(CN)_4N]^-$, on parameter h in the DE-correction in ωLH23td

h	A_{iso}					A_{dip}	$\langle S^2 \rangle$
	1s	2s	3s	3s/2s	total		
1.0	-6.7	-605.1	328.9	-0.54	-272.4	-118.0	0.911
2.0	-5.2	-582.9	305.5	-0.52	-277.9	-119.4	0.888
3.0	-3.7	-557.1	279.7	-0.50	-281.8	-121.4	0.863
4.0	-2.2	-526.2	250.7	-0.48	-283.2	-123.9	0.836
5.0	-0.7	-489.7	218.1	-0.45	-281.8	-127.2	0.809
6.0	0.6	-447.7	181.9	-0.41	-277.5	-131.3	0.784
7.0	1.8	-405.8	145.9	-0.36	-272.4	-135.4	0.766
8.0	2.9	-378.5	118.9	-0.31	-273.1	-137.9	0.759
9.0	3.8	-366.3	101.2	-0.28	-279.0	-138.7	0.757
10.0	4.8	-357.5	85.8	-0.24	-285.4	-139.0	0.756
11.0	5.6	-350.0	70.8	-0.20	-292.4	-139.2	0.756
12.0	6.5	-345.4	58.5	-0.17	-299.7	-139.3	0.756
Exp. [S5]					-276	-122.4	0.750

Table S13. Dependence of CSSP, A_{iso}, A_{dip}, and $\langle S^2 \rangle$ for cluster-embedded $[Mn(CN)_5NO]^{2-}$ on parameter h in the DE-correction.

	h	A_{iso}					A_{dip}	$\langle S^2 \rangle$
		1s	2s	3s	3s/2s	total		
ωLH23td	6.0	-0.6	-499.8	202.6	-0.41	-220.4	-100.5	0.873
	7.0	0.7	-467.5	171.0	-0.37	-225.0	-105.7	0.839
	8.0	2.0	-419.5	128.9	-0.31	-226.2	-113.6	0.798
	9.0	3.0	-376.4	90.3	-0.24	-227.4	-120.6	0.773
	10.0	3.9	-358.7	68.3	-0.19	-233.3	-123.2	0.766
	11.0	4.8	-349.7	51.9	-0.15	-240.9	-124.2	0.765
	12.0	5.5	-345.1	38.8	-0.11	-249.1	-124.5	0.764
ωLH23tdB	6.0	7.1	-420.5	149.3	-0.35	-196.0	-114.4	0.791
	7.0	9.1	-385.8	116.0	-0.30	-198.9	-119.9	0.773
	8.0	11.0	-365.7	92.2	-0.25	-204.3	-122.8	0.766
	9.0	12.9	-351.9	72.6	-0.21	-210.5	-124.6	0.762
	10.0	14.7	-342.3	55.8	-0.16	-217.0	-125.7	0.761
	11.0	16.5	-336.1	40.9	-0.12	-224.4	-126.2	0.760
	12.0	18.1	-331.1	27.7	-0.08	-231.4	-126.5	0.760
Exp. [S6]						-219.5	115.2	0.750

References

(S1) Buschmann, W. E.; Arif, A. M.; Miller, J. S. Structure and Properties of Tetracyanomanganate (II), $[Mn^{II}(CN)_4]^{2-}$, The First Paramagnetic Tetrahedral Cyanometalate Complex. *Angew. Chem., Int. Ed. Engl.* **1998**, *37*, 781–783.

(S2) Ferrante, R. F.; Wilkerson, J. L.; Graham, W. R. M.; Weltner Jr., W. ESR spectra of the MnO, MnO_2, MnO_3, and MnO_4 molecules at 4 K. *J. Chem. Phys.* **1977**, *67*, 5904–5913.

(S3) Munzarova, M.; Kaupp, M. A critical validation of density functional and coupled-cluster approaches for the calculation of EPR hyperfine coupling constants in transition metal complexes. *J. Phys. Chem. A* **1999**, *103*, 9966–9983.

(S4) Schattenberg, C. J.; Maier, T. M.; Kaupp, M. Lessons from the Spin-Polarization/Spin-Contamination Dilemma of Transition-Metal Hyperfine Couplings for the Construction of Exchange-Correlation Functionals. *J. Chem. Theory Comput.* **2018**, *14*, 5653–5672.

(S5) Bendix, J.; Meyer, K.; Weyhermueller, T.; Bill, E.; Metzler-Nolte, N.; Wieghardt, K. Nitridocyanometalates of Cr^{V}, Mn^{V}, and Mn^{VI}. *Inorg. Chem.* **1998**, *37*, 1767–1775.

(S6) Manoharan, P. T.; Gray, H. B. Electronic Structures of Metal Pentacyanonitrosyls. *Inorg. Chem.* **1966**, *5*, 823–839.
